# Clinical Phenotypes in Hypertension: A Data-Driven Approach to Risk Stratification

**DOI:** 10.1161/HYPERTENSIONAHA.125.25187

**Published:** 2025-12-11

**Authors:** Elisa Rauseo, Ahmed M. Salih, Jackie Cooper, Musa Abdulkareem, Christopher R.S. Banerji, Sucharitha Chadalavada, Hafiz Naderi, Patricia B Munroe, Anthony Mathur, Nay Aung, Gregory G. Slabaugh, Steffen E. Petersen

**Affiliations:** 1 William Harvey Research Institute, NIHR Barts Biomedical Research Centre (E.R., A.M.S., J.C., M.A., S.C., H.N., P.B.M., N.A., S.E.P.), Queen Mary University London, Charterhouse Square, United Kingdom.; 2 Digital Environment Research Institute (E.R., G.G.S.), Queen Mary University London, Charterhouse Square, United Kingdom.; 3 Centre for Cardiovascular Medicine and Devices, William Harvey Research Institute (A.M.), Queen Mary University London, Charterhouse Square, United Kingdom.; 4 NIHR Barts Biomedical Research Centre (A.M.), Queen Mary University London, Charterhouse Square, United Kingdom.; 5 School of Electronic Engineering & Computer Science (G.G.S.), Queen Mary University London, Charterhouse Square, United Kingdom.; 6 Barts Heart Centre, St Bartholomew’s Hospital, Barts Health NHS Trust, West Smithfield, EC1A 7BE, United Kingdom (E.R., A.M.S., M.A., S.C., H.N., A.M., N.A., S.E.P.).; 7 Department of Population Health Sciences, University of Leicester, United Kingdom (A.M.S.).; 8 PRIME Lab, Scientific Research Center, University of Zakho, Kurdistan Region, Iraq (A.M.S.).; 9 The Alan Turing Institute, London, United Kingdom (C.R.S.B.).; 10 King’s Comprehensive Cancer Centre, King’s College London, United Kingdom (C.R.S.B.).; 11 University College London NHS Trust, United Kingdom (C.R.S.B.).

**Keywords:** atherosclerosis, atrial fibrillation, heart failure, magnetic resonance imaging, phenotype

## Abstract

**BACKGROUND:**

Hypertension is a major contributor to cardiovascular morbidity and mortality. Its heterogeneity complicates risk stratification. Unsupervised machine learning can uncover risk profiles and refine preventative strategies. This study applied a data-driven approach to identify clinical phenotypes of hypertension, examine their associations with cardiovascular imaging characteristics and adverse outcomes, and assess the mediating role of cardiac imaging features in these associations.

**METHODS:**

Fourteen thousand eight hundred forty UK Biobank participants with diagnosed hypertension and cardiovascular magnetic resonance imaging were analyzed. K-means clustering was applied to 77 clinical variables. Associations with incident heart failure, atrial fibrillation, atherosclerotic events, all-cause mortality, and major adverse cardiovascular events were examined and adjusted for cardiovascular risk factors. Mediation analyses assessed the role of cardiovascular imaging features in the association between clusters and outcomes.

**RESULTS:**

Three clusters emerged. Cluster 1, predominantly female with the most favorable metabolic profile, had the lowest risk. Cluster 2, predominantly male with the highest atherosclerosis burden, carried the greatest risk for all adverse events, independent of cardiovascular risk factors. They showed severe cardiac remodeling, impaired cardiac mechanisms, and global left atrial dysfunction. Cluster 3 had a profile resembling metabolic syndrome, with moderate risk for atrial fibrillation and all-cause death (hazard ratio, 1.65 and 1.58; *P*<0.05). Although in cluster 2 the risk was largely mediated by left ventricular hypertrophy, in cluster 3 its role was attenuated and more evenly balanced with left atrial dysfunction.

**CONCLUSIONS:**

Clustering analysis identified distinct hypertension phenotypes with specific risk profiles, suggesting potential for improved stratification and more tailored treatment approaches.

**Novelty and Relevance**

**What Is New?**

Unsupervised clustering identified 3 distinct hypertension phenotypes within a large UK Biobank population. These phenotypes differed in clinical profiles, cardiovascular imaging features, and risks of adverse events. Cardiovascular imaging traits played differing roles in linking clusters to outcomes, suggesting varied underlying mechanisms.

**What Is Relevant?**

Standard risk models may overlook meaningful patient subgroups.

Clustering captures hidden heterogeneity in hypertension. Integrated clinical–imaging phenotyping supports more precise risk stratification.

**Clinical/Pathophysiological Implications.**

Distinct clusters reflect diverse pathways leading to hypertensive heart disease and adverse outcomes. Understanding differential imaging patterns may guide more tailored prevention strategies.

These findings support future research toward personalized hypertension care.

Essential hypertension is a major modifiable risk factor contributing to global mortality and morbidity.^1^ Chronic hypertension can impact target organs, leading to structural and functional changes in the heart, arteries, kidneys, and brain. Without treatment, these changes may worsen, resulting in severe complications, such as heart failure (HF), myocardial infarction (MI), atrial fibrillation (AF), stroke, cognitive decline, and kidney diseases. These chronic conditions can significantly impact patients’ outcomes, highlighting the importance of effective hypertension risk assessment and preventive measures.^1^

Although blood pressure reduction remains central to hypertension management, multiple factors influence disease trajectory, treatment response, and outcomes. These include environmental, socioeconomic, psychological, demographic, lifestyle, genetic, and systemic mechanisms across multiple organs. This heterogeneity complicates risk stratification and management. Traditional risk stratification models rely largely on blood pressure thresholds and traditional risk factors to predict 10-year cardiovascular disease (CVD) risk.^1^ Although guidelines now recommend integrating nontraditional modifiers (eg, socioeconomic status) to refine risk assessment and guide management, these approaches fail to capture hypertension heterogeneity, leaving some patients at high risk despite optimal blood pressure control, while others experience adverse outcomes even at lower blood pressure levels.^1,2^ This underscores the need for a more comprehensive approach that accounts for the full spectrum of contributing factors and enables personalized risk-based management.

Clustering is an unsupervised machine-learning method that identifies patterns and subgroups within heterogeneous populations. It has improved phenotyping and risk stratification for several CVDs, including HF, and provided pathophysiological insights.^3,4^ Previous clustering studies in hypertension have identified clinically significant phenotypes, broadening classification beyond blood pressure values and offering further insights for guiding personalized preventative measures and treatments.^5–9^ However, most previous studies had limited sample sizes, few clinical variables for risk stratification, and lacked links between clinical phenotypes, cardiovascular imaging changes, and long-term outcomes.

To address the challenges of risk stratification in hypertension, this study adopts a data-driven approach within a large UK Biobank cohort. Specifically, it aims to identify distinct clinical phenotypes using unsupervised machine learning, examine their associations with cardiovascular imaging characteristics and adverse outcomes, and explore the potential mediating role of cardiac imaging features in these associations. Although observational and hypothesis-generating, this approach may help uncover mechanisms underlying heterogeneity in hypertension and support the development of more personalized strategies by identifying patient subgroups with differing risk profiles and imaging characteristics.

## Methods

### Data Availability

This study is based on data from the UK Biobank (application number 2964). For more details on the access procedure, see the UK Biobank website (http://www.ukbiobank.ac.uk/register-apply/).

The UK Biobank is a large, population-based cohort study that recruited over 500 000 participants aged 40 to 69 years between 2006 and 2010 from across the United Kingdom.^10^ Baseline information includes socio-demographics, lifestyle, medical history, genetics, physical measures, and health-related outcomes. As part of the ongoing imaging substudy, >50 000 participants underwent cardiovascular magnetic resonance (CMR) imaging.

All participants provided written informed consent, and the study complies with the Declaration of Helsinki. Ethical approval was granted by the National Health System (NHS) National Research Ethics Service (Ref 11/NW/0382; renewed under Ref 21/NW/0157).

### Study Population

The study population included UK Biobank participants with a confirmed diagnosis of hypertension, defined through a combination of International Classification of Diseases (ICD) codes, self-reported disease, doctor-diagnosed conditions, and the use of antihypertensive medications up until the time of the first imaging visit. Blood pressure values alone were not used to define hypertension status, as isolated elevations may not reflect established disease. To focus on individuals with hypertension without advanced cardiac pathology, we excluded those with a history of HF at the time of imaging. This approach aimed to capture a clinically relevant hypertension cohort, free from overt HF, to explore phenotypic differences and their association with cardiovascular risk. The specific UK Biobank field codes used to define the study population are detailed in Table S1.

### Phenotypic Domains

Baseline characteristics of the study population, including socio-demographic factors, lifestyle habits, physical attributes, ECG parameters, and laboratory data, were collected and used as phenotypic domains for clustering analysis. The full list of phenotypic features scrutinized for clustering, with corresponding UK Biobank data fields, is reported in Table S2. A more detailed description of how the phenotypic features were calculated is provided in a previous publication.^3^

### Imaging Parameters

CMR-derived features, not included in clustering analysis, were utilized in posthoc analyses to validate clusters and assess their influence on outcomes. A detailed description of CMR acquisition and analysis is provided in Imaging Parameters in Supplemental Methods.

The following CMR metrics, indexed to body surface area where appropriate, were analyzed: left ventricular (LV) and right ventricular volumes, stroke volumes and ejection fractions; LV mass, LV maximum wall thickness, mass-to-volume ratio, LV global function index and native myocardial T1 (mid-short-axis). Cardiac mechanics included global longitudinal strain (GLS), global circumferential strain (GCS), global radial strain (GRS), and torsion.^11,12^ GRS is positive, while GLS and GCS are negative; all strain metrics are reported as absolute values for consistency.

Left atrial (LA) volumes included indexed maximum, minimum, and preatrial contraction volumes. From these, the total, passive, and active LA emptying fraction were derived, reflecting reservoir, conduit, and booster pump functions. LA expansion index, reflecting LV reservoir function, was also calculated.

Arterial function was assessed using total arterial compliance, aortic distensibility, and systemic vascular resistance, to capture changes in ventricular-arterial coupling relevant to hypertension.

### Ascertainment of Outcomes

Clinical outcomes occurring after the imaging visit (incident events) were identified using specific UK Biobank fields (Table S3). Survival analyses involved censoring individuals based on the event date, date of death, or end of follow-up (October 31, 2022), whichever came first. The clinical end points of interest included all-cause HF, all-cause mortality, AF, and atherosclerotic events. All-cause HF comprised HF, pulmonary edema, or any cardiomyopathy as a possible cause.^13^ Atherosclerotic events included nonfatal MI, stroke, or peripheral artery disease. A composite of major adverse cardiovascular events (MACE) was also assessed, including HF, AF, atherosclerotic events, or CVD mortality.

### Analysis Workflow

The overall analysis comprised 3 main stages: (1) data preparation, (2) clustering analysis, and (3) posthoc statistical analyses. Full methodological details are provided in Analysis Workflow in Supplemental Methods.

Briefly, of 79 candidate features (Table S2), 77 (<20% missingness) were included. Missing values were imputed, and the cleaned data set was used for clustering. Several clustering approaches were explored and compared for performance, computational efficiency, and clinical interpretability. Factorial Analysis of Mixed Data followed by K-Means on the full data set was selected. Forty Factorial Analysis of Mixed Data-derived components (>80% variance explained) were retained, and a 3-cluster solution was chosen using the elbow method, Silhouette score, and clinical interpretability. Stability was confirmed with bootstrapping and the adjusted Rand index. For interpretation, Shapley Additive Explanations were applied to a supervised classifier trained with the 40 components, which identified and ranked the 32 most important features contributing to cluster assignment (Table 1).

**Table 1.**
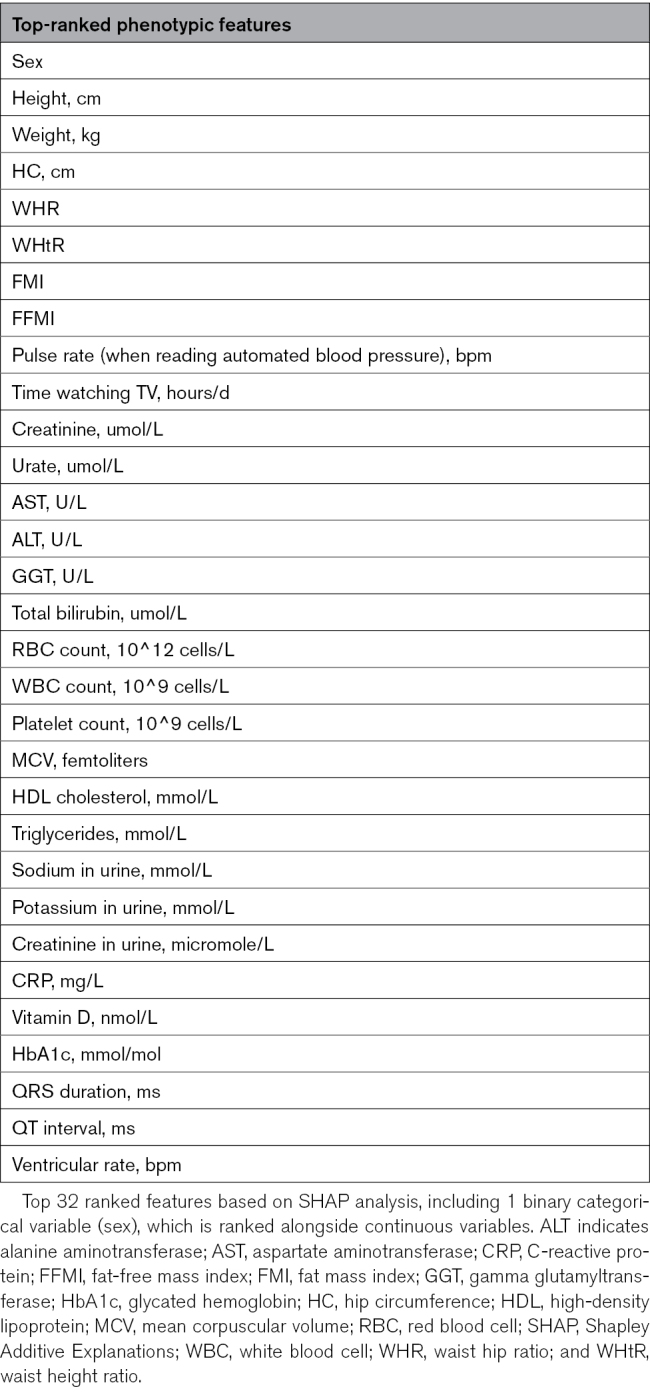
Top-Ranked Phenotypic Features Contributing to Clustering

Posthoc analyses examined associations of clusters with outcomes, cardiac structure/function, and mediation by CMR features. Cox proportional hazards models tested associations with incident outcomes, excluding participants with the corresponding prevalent disease. All models were adjusted for diabetes, hypercholesterolemia, and prior CVD, with the latter omitted when prevalent cases were already excluded. To account for competing risks, non-CVD death (<2%) was modeled separately. As a sensitivity analysis, models were repeated after excluding participants with <6 months of follow-up to reduce potential reverse causality. Model performance was assessed with Nagelkerke pseudo-R² and concordance index (C-statistic) across cluster-only, covariate-only, and fully adjusted models.

Multivariable regression assessed associations between clusters and CMR traits, adjusting for the same covariates used in the outcome analyses. To confirm that CMR-cluster associations did not reflect sequelae of preexisting disease, we repeated the analyses after excluding participants with prior cardiovascular conditions and adjusting for the residual risk factors (diabetes, high cholesterol). Finally, mediation analyses using logistic regression tested whether specific CMR features mediated the cluster-outcome associations. These traits were selected as mediators given their role as markers of hypertensive target-organ damage and recognized position along the causal pathway from high blood pressure to cardiovascular events. We hypothesized that their varying levels across clusters could help explain differences in outcome risk, reflecting heterogeneous contributions to cardiovascular burden. Each model tested 1 continuous CMR feature as a mediator between cluster and outcome, adjusted for the same covariates as the Cox models. The proportion mediated was calculated as the ratio of the indirect to total effect,^14^ allowing estimation of how much of the cluster-outcome association could be explained by individual CMR features. Since each model was run independently, mediated proportions are not additive.

Two sensitivity analyses excluding cardiomyopathy and participants with long baseline–CMR intervals were finally performed to confirm robustness to disease and timing effects.

### Statistical Analysis

Clusters were compared on clinical characteristics using χ^2^ tests for categorical variables and ANOVA (or Kruskal-Wallis) for continuous variables. Pairwise comparisons used *t* tests or Wilcoxon rank-sum tests for numeric data and χ^2^ tests for categorical data. A 2-sided *P*<0.05 with Bonferroni correction, where appropriate, was considered significant. Analyses were conducted using Python 3.8.10 (Python Software Foundation, DE) and Scikit-learn 0.23.2.^15^

## Results

### Study Population Characteristics

The hypertension cohort (n=14 840) was predominantly middle-aged (66.56±7.24 years), White (97%), and included 42% of females. Cardiovascular risk factors were prevalent, including hypercholesterolemia (53%), smoking (17%), and diabetes (12%). The prevalence of MI was ≈11% (Table S4). The population exhibited, overall, poorly controlled hypertension (systolic blood pressure [SBP], 147.1±18.3 mm Hg; diastolic blood pressure, 81.5±10.3 mm Hg). Participants were followed for a median of 51.7 months (interquartile range, 41.8–68.1), with a minimum of 0.2 months and a maximum of 102 months. Over this period, 591(3.98%) subjects developed HF, 778 (5.24%) experienced atherosclerotic events, 366 (2.46%) were diagnosed with AF, and 244 (1.64%) died. The overall incidence of MACE was 8.84% (n=1312).

### Identifying Distinct Clusters

Clustering stratified the individuals with hypertension into 3 phenotypically distinct groups, as determined by the elbow method and supported by Silhouette scores and clinical interpretability (Analysis Workflow in Supplemental Methods). Cluster stability was high (adjusted rand index=0.80), indicating strong agreement across resamplings. The resulting groups showed significant differences across a wide range of clinical, behavioral, laboratory, and ECG characteristics (Table 2; Tables S5 and S6).

**Table 2.**
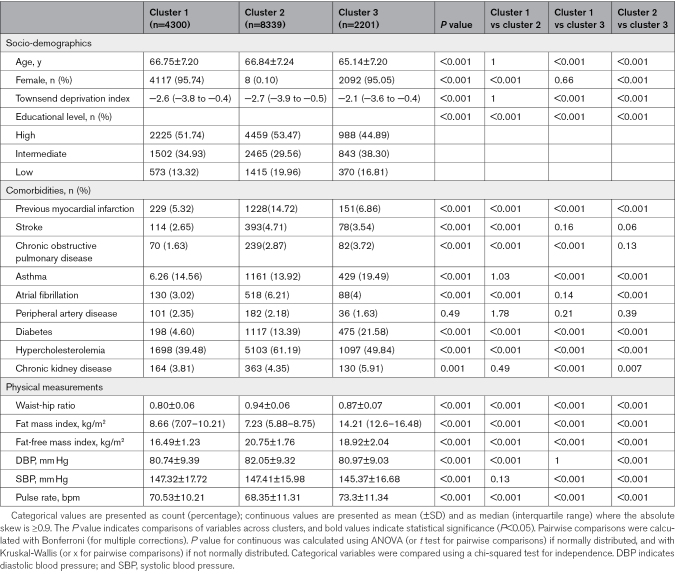
Baseline Clinical Characteristics Stratified by Clusters

Cluster 1 (n=4300) was predominantly female (95.7%) with leaner body composition, lower diabetes (4.6%) and hypercholesterolemia (39.5%) rates, and moderate smoking history (35.4%). This group exhibited better lifestyle habits, a more favorable metabolic profile (eg, higher high-density lipoprotein and lower triglycerides), and decreased systemic inflammation (C-reactive protein). They had distinct ECG features, such as a shorter QRS duration than cluster 2 and a lower resting heart rate than cluster 3.

Cluster 2 (n=8339) was mainly male (99.9%) and exhibited significant CVD burden, including MI (14.7%) and AF (6.2%). This group had a high waist-to-hip ratio but a lower fat mass index than cluster 3. They also had the lowest low-density lipoprotein and high-density lipoprotein cholesterol levels and signs of subclinical kidney dysfunction. Lifestyle habits included high smoking rates (49.4%) and alcohol and processed food consumption. Their ECG profile showed the longest QRS duration (90 ms) and the lowest resting pulse rate.

Cluster 3 (n=2201) was predominantly female, like cluster 1, but younger (65.14±7.20 years), with the highest central adiposity, and the greatest prevalence of diabetes (21.6%). They had lower physical activity, greater socioeconomic deprivation, and the highest systemic inflammatory markers. This group showed a higher resting heart rate (64 bpm) and shorter QRS duration (86 ms) than cluster 2. With regard to the blood pressure values, cluster 3 showed the lowest SBP (145.37 mm Hg), while cluster 2 displayed the highest diastolic blood pressure (82.05 mm Hg) among the groups.

**Figure 1.**
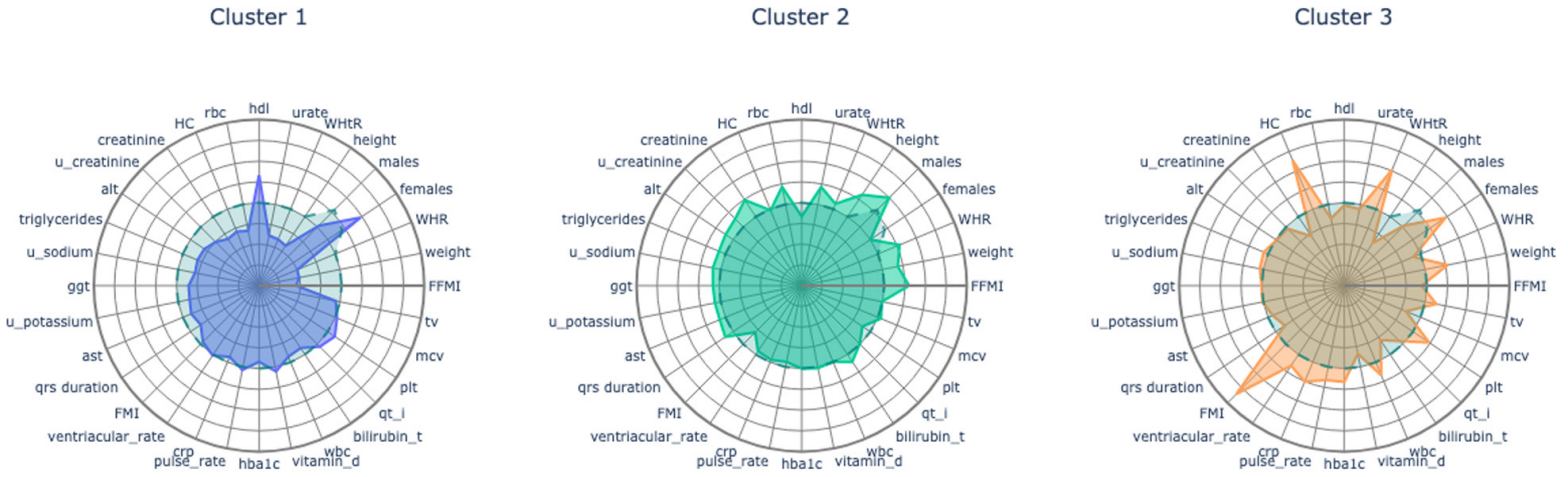
**Radar charts showing the top 32 features contributing to clustering.** Radar plots summarizing the top 32 (scaled) features contributing to clustering obtained from Shapley Additive Explanations (SHAP) compared with their average value for the entire cohort (indicated by the dashed blue line). alt indicates alanine aminotransferase; ast, aspartate aminotransferase; FFMI, fat free mass index; FMI, fat mass index; ggt, gamma glutamyltransferase; HbA1c, glycated hemoglobin; HC, hip circumference; mcv, mean corpuscular volume; plt, platelet; rbc, red blood cell; tv, time spent on television; wbc, white blood cell; WHR, waist hip ratio; and WHTr, waist height ratio.

The top 32 Shapley Additive Explanations features identified for effective clustering summarized diverse clinical phenotypes (Figure 1). They included body composition, metabolic markers, cardiovascular characteristics, and lifestyle habits, reflecting each cluster’s risk profile. This confirms Shapley Additive Explanations as a valuable tool for capturing core characteristics and enhancing interpretability while facilitating meaningful comparisons between clusters.

### Associations With Clinical Outcomes

Kaplan-Meier analyses revealed significantly different survival trajectories across clusters for all adverse events (Figure S1; multivariate log-rank test *P*<0.005). Cluster 1 exhibited the lowest risk, cluster 2 the highest, and cluster 3 an intermediate profile.

Cox models confirmed these findings (Table 3). After covariate adjustments, cluster 2 consistently showed the greatest risk versus cluster 1, with the highest hazard ratios (HR) for atherosclerotic events (HR, 1.91 [95% CI, 1.53–2.38]; *P*<0.005), AF (HR, 1.89 [95% CI, 1.43–2.51]; *P*<0.005), and HF (HR, 1.77 [95% CI, 1.42–2.20]; *P*<0.005). Cluster 3 showed moderate risk for AF (HR, 1.65 [95% CI, 1.14–2.37]; *P*<0.05) and mortality (HR, 1.58 [95% CI, 1.01–2.47]; *P*<0.05). Non-CVD death was not significantly associated with clusters, confirming competing risks did not bias cause-specific hazard estimates.

**Table 3.**
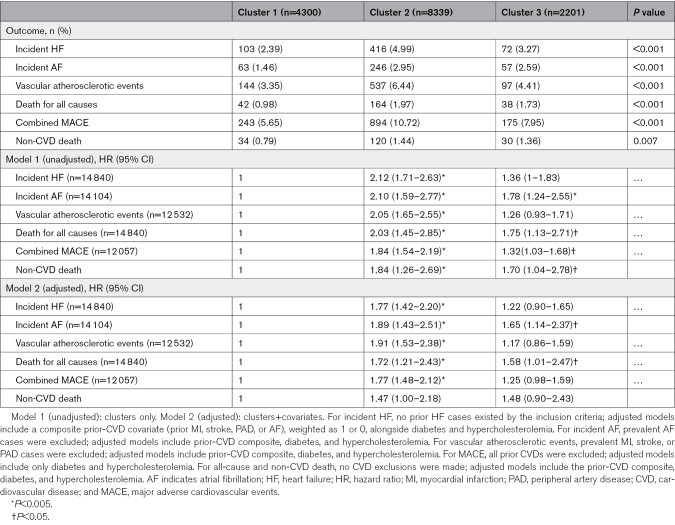
Association of Clusters With Adverse Outcomes on Cox Proportional Hazards Analysis

Sensitivity analyses excluding follow-up <6 months confirmed the overall patterns. Cluster 2 associations remained the same, while in cluster 3, AF risk persisted, but mortality lost significance (Table S7), supporting robustness and temporal validity.

Pseudo-R² values were low across all models, as expected in time-to-event analyses due to censoring and low event rates. However, adding cluster membership to covariate-only models consistently improved both pseudo-R² and concordance (Table S8), with the greatest gains for atherosclerotic events, MACE, and AF. This suggests cluster membership adds modest prognostic information beyond baseline covariates. Still, given the limited variance explained (Nagelkerke pseudo-R² <0.02), clusters should be viewed as complementary tools that modestly enhance risk stratification.

### Associations With CMR Metrics

**Figure 2.**
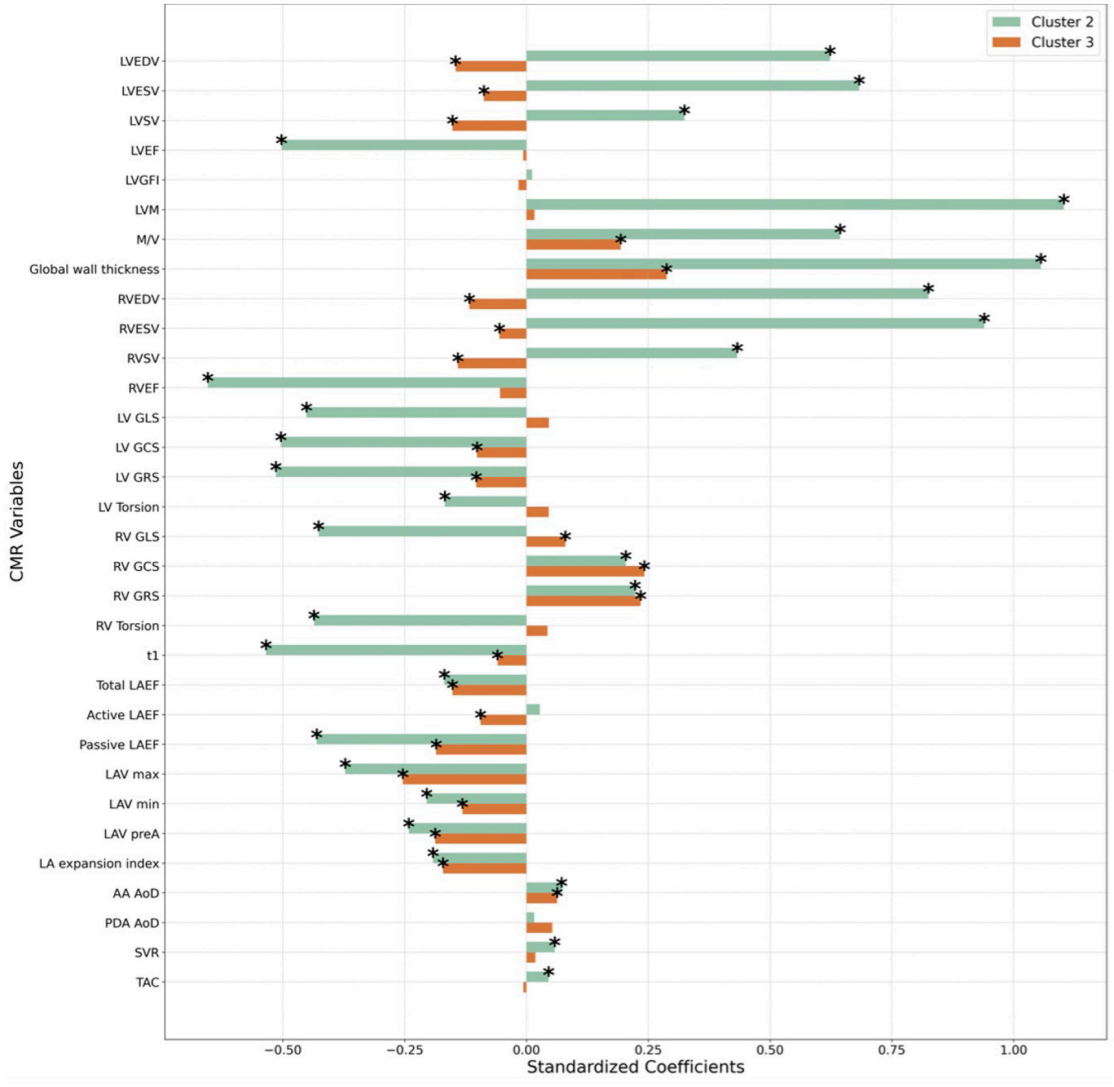
**Associations between cardiovascular magnetic resonance (CMR) metrics and clustering.** The bars represent standardized beta coefficients from regression models, indicating the magnitude and direction of changes in each CMR metric when comparing clusters 2 and 3 to cluster 1. Positive coefficients suggest higher values of the respective CMR metric in clusters 2 or 3 compared with cluster 1, while negative coefficients indicate lower values relative to the reference group. Only significant associations after Bonferroni correction for multiple testing are displayed. Asterisks (*) indicate statistically significant associations within each group. AA indicates ascending aorta; AoD, aortic distensibility; DA, descending aorta; GCS, global circumferential strain; GLS, global longitudinal strain; GRS, global radial strain; LAEF, left atrial emptying fraction; LAV, left atrial volume; LVEDV, left ventricular end-diastolic volume; LVEF, left ventricular ejection fraction; LVM, left ventricular mass; LVESV, left ventricular end-systolic volume; LVGFI, left ventricle global function index; LVSV, left ventricular stroke volume; M/V, LV mass-to-volume ratio; RVEDV, right ventricular end-diastolic volume; RVEF, right ventricular ejection fraction; RVESV, right ventricular end-systolic volume; RVSV, right ventricular stroke volume; SVR, systemic vascular resistance; and TAC, total arterial compliance.

CMR analysis revealed significant differences across the clusters (Figure 2; Table S9), with cluster 1 showing the most favorable cardiovascular imaging features. Cluster 2 was linked to the largest biventricular volumes, greatest cardiac remodeling (highest mass-to-volume ratio), and LV hypertrophy, along with impaired biventricular systolic function. It also had the poorest LA function (primarily passive LA emptying fraction), LV mechanics (GLS, GRS, GCS), as well as right ventricular longitudinal strain. Despite minimal arterial stiffening (the highest total arterial compliance and aortic distensibility), it showed the greatest systemic vascular resistance. Cluster 3 had smaller biventricular volumes than the reference groups, milder LV remodeling, and LV hypertrophy compared with cluster 2, along with less reduction in LV mechanics (GCS and GRS) and better right ventricular strains. They also showed a milder reduction in LA functions, including the LA booster pump function. These associations remained consistent after excluding participants with prior cardiovascular conditions and adjusting for residual risk factors, confirming that CMR-cluster differences were not driven by disease sequelae (Figure S2).

### Mediation Role of CMR Metrics in the Association Between Cluster and Outcomes

The proportion mediated, expressed as a percentage, reflects the extent to which each individual CMR metric statistically explains the association between cluster membership and each clinical outcome (indirect effect), after adjusting for baseline vascular risk factors and prevalent conditions.

**Figure 3.**
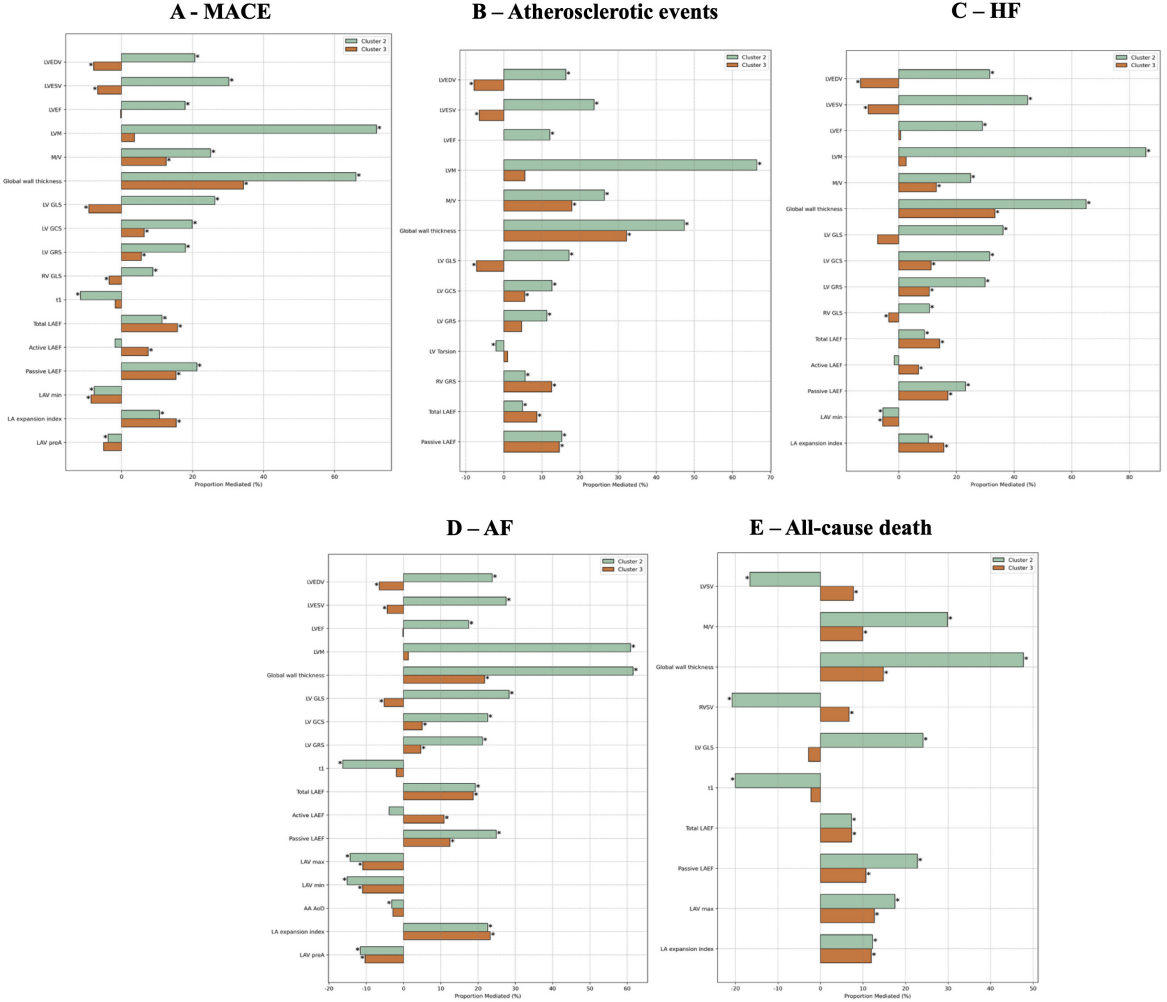
**Proportion of risk mediated by cardiovascular magnetic resonance (CMR) features across clusters for each cardiovascular outcome.** The figure shows the proportion of the total effect of clustering on each outcome that is mediated through individual CMR metrics, expressed as a percentage. **A**, Major adverse cardiovascular event (MACE), (**B**) atherosclerotic events, (**C**) heart failure (HF), (**D**) atrial fibrillation (AF), (**E**) all-cause death. Positive values indicate mediation, where the CMR variable explains part of the association between clustering and the outcome. Negative values suggest a suppressor effect, strengthening the direct association. Cluster 2 (higher-risk) and cluster 3 (intermediate-risk) are compared with cluster 1 (lowest-risk reference). Asterisks (*) indicate statistically significant mediation effects. AA indicates ascending aorta; AoD, aortic distensibility; GCS, global circumferential strain; GLS, global longitudinal strain; GRS, global radial strain; LA, left atrial; LAEF, left atrial emptying fraction; LAV, left atrial volume; LVEDV, left ventricular end-diastolic volume; LVEF, left ventricular ejection fraction; LVESV, left ventricular end-systolic volume; LVM, left ventricular mass; M/V, mass-to-volume ratio; and RVSV, right ventricular stroke volume.

For MACE (Figure 3A), several CMR features significantly mediated risk in cluster 2. The largest proportions mediated were observed for LV hypertrophy (eg, LV mass, 72%; global wall thickness, 65%), geometric remodeling (mass-to-volume ratio, 25%), impaired LV mechanics (GLS, 26%), altered passive LA emptying fraction (21%), LV ejection fraction (18%), and LV volumes. Myocardial native T1 acted as a suppressor, with a proportion mediated of –11%. In cluster 3, mediation effects were smaller, with the highest proportion mediated by LV maximum wall thickness (34%) but not LV mass, followed by indices of LA function, including total (16%), passive (15%), expansion index (15%), and active (8%) LA emptying fraction, as well as geometric remodeling (mass-to-volume ratio, 13%).

Mediation patterns in atherosclerotic events and HF (Figure 3B and C) largely resembled those seen in MACE across both clusters. However, mediation effects were more pronounced in HF, particularly in cluster 2, where LV hypertrophy and cardiac remodeling were dominant mediators.

For AF (Figure 3D), mediation patterns broadly mirrored those seen in MACE, but indices of LA function played a more prominent role in AF risk, as multiple LA metrics were significant mediators in both clusters, with higher proportions mediated than in MACE. In cluster 2, additional CMR metrics emerged as suppressors, including myocardial native T1 and aortic distensibility (−19% and −5%, respectively).

Fewer CMR metrics significantly mediated mortality risk (Figure 3E). In cluster 2, significant indirect effects were observed for global wall thickness, cardiac remodeling, LV GLS, and multiple LA function indices, whereas LV mass was not a significant mediator. Conversely, right ventricular and LV stroke volumes, along with native T1, acted as suppressors. These findings suggest that atrial dysfunction and LV remodeling are key contributors to mortality in this cluster. In cluster 3, mediation was limited to global wall thickness and LA function metrics, with similar proportions mediated for both.

### Sensitivity Analyses

Both sensitivity analyses, excluding cardiomyopathy cases and participants with long baseline-CMR intervals, yielded consistent results, confirming robustness (Supplemental Results).

## Discussion

In this large UK Biobank cohort, unsupervised clustering of clinically available data identified 3 hypertension phenotypes with distinct risk profiles, cardiovascular imaging features, and outcomes despite modest blood pressure differences. The cardiovascular changes played varying roles in mediating risk, underscoring heterogeneity among individuals with HTN and the potential of clustering for refining risk stratification (Graphical Abstract).

Although this was an exploratory, hypothesis-generating study that did not formally compare cluster membership to traditional risk scores, clusters still provided additional prognostic information. When added to Cox models already adjusted for conventional cardiovascular risk factors and comorbidities, cluster membership modestly improved concordance and pseudo-R². These findings suggest that data-driven phenotypes may capture dimensions of risk not fully reflected by standard clinical variables, offering complementary value for understanding and stratifying heterogeneity in hypertension.

### Three Distinct Phenotypes of Hypertension

Cluster 1 represented the most favorable hypertension phenotype, mainly females with healthier metabolisms and lifestyles. Despite the lowest aortic compliance, likely reflecting female predominance, they showed minimal cardiac remodeling.^16^ This milder profile resulted in better clinical outcomes, categorizing this group as low risk.

Cluster 2, predominantly male, exhibited an advanced atherosclerotic profile, with high MI prevalence, low high-density lipoprotein, and target organ damage (eg, poor kidney function). Total cholesterol and low-density lipoprotein were low, likely from secondary prevention. This phenotype showed marked cardiac remodeling, with LV hypertrophy, larger ventricular volumes, impaired LV mechanics (but preserved ejection fraction), and LA dysfunction. Although SBP was similar to cluster 1, cluster 2 had higher diastolic blood pressure and systemic vascular resistance, indicating more severe vascular disease. Combined with LV hypertrophy and atherosclerosis, these changes likely drove greater LV dysfunction and heightened risk of adverse events,^11,17^ consistent with prior evidence in middle-aged males.^18,19^

Cluster 3, mainly female, exhibited features of metabolic syndrome (MetS)—central adiposity, poor glucose control, systemic inflammation—and sociocultural challenges. Although LV hypertrophy and atrial dysfunction were milder than in cluster 2, LA impairment was more extensive, affecting all functional phases. This group had lower SBP and better aortic compliance than cluster 1, suggesting a relatively favorable vascular profile.^20^ However, they carried intermediate AF and mortality risk. This aligns with evidence that MetS promotes AF through atrial remodeling, cardiac autonomic changes, and systemic inflammation.^21,22^ Women may be more vulnerable due to postmenopausal metabolic shifts, genetic and sociocultural factors.^22,23^ Although obesity, diabetes, and dyslipidemia can each contribute to cardiac remodeling, their coexistence can amplify these effects, markedly increasing AF susceptibility.^21,22^ This may explain the elevated AF risk in this female-dominant group, where multiple MetS components were present.

Interestingly, despite LA dysfunction in both cluster 2 and 3, neither showed increased LA volumes, included indexed, suggesting functional deterioration may precede dilatation or LV hypertrophy, particularly in cluster 3. This emphasizes LA function as an earlier predictor of AF risk in hypertension before LV hypertrophy.^24–28^

The sensitivity analysis showed that including subjects with subclinical cardiomyopathies did not significantly change the clustering results but slightly overestimated the mediating effects of LV remodeling on mortality in cluster 2, and attenuated the roles of GLS in cluster 3. These findings confirm that clustering-derived risk profiles are robust and reflect true underlying phenotypes.

### Imaging Insights Into Pathophysiological Mechanisms of Risks

Analyzing the imaging findings in relation to outcomes provided key insights into potential pathophysiological mechanisms underlying each phenotype of hypertension. Clusters in the order 1, 3, and 2 showed a stepwise increase in concentric LV hypertrophy, worsening LV mechanics, and declining diastolic function (lower LA expansion index), mirroring their escalating risk of adverse events. While these changes align with hypertensive heart disease progression, the interplay of comorbidities uniquely shaped these patterns.^29,30^

The significant LV remodeling and global impairment in LV mechanics (including GLS, GCS, and GRS) observed in cluster 2 resemble the HF with preserved ejection fraction substrate.^9^ The LA dysfunction, particularly in reservoir and conduit function, is primarily driven by the high comorbidity burden, especially prevalent MI and systemic organ dysfunction, which further increases cardiovascular risk.^19,27,28,31,32^

The cardiac changes observed in cluster 3, featuring less severe LV remodeling but LA dysfunction across all phases (reservoir, conduit, and contractile), likely reflect the metabolic and inflammatory burden of MetS, which promotes atrial remodeling before the emergence of significant LV changes, thus creating an arrhythmic substrate for AF risk.^24–28,30^ Similar patterns of LA dysfunction have been reported in specific hypertension populations and in cohorts susceptible to AF with elevated metabolic risk factors.^26,28,33^ This underscores how MetS-induced systemic pathways contribute to atrial remodeling, thereby increasing AF susceptibility.

Both clusters 2 and 3 showed an increased AF risk, confirming the well-established bidirectional hypertension AF connection, though driven by 2 distinct mechanisms.^34^ In cluster 2, hypertension-induced remodeling and coexisting atherosclerosis created a pro-HF environment that heightened AF susceptibility, reinforcing evidence that HF with preserved ejection fraction and AF share a common myocardial disease substrate.^35^ Conversely, in cluster 3, MetS-related metabolic and inflammatory factors primarily induced atrial changes that made individuals more prone to AF, emphasizing the necessity for targeted risk-factor management in this group.^28,33^

The mediation analyses further clarified how these phenotypic differences influenced adverse events. In cluster 2, where LV remodeling was most pronounced, LV hypertrophy and LV mechanical dysfunction emerged as key mediators of clinical risk, especially for HF. LA dysfunction, instead, played a greater role in all-cause mortality. These findings confirm the prognostic impact of LV hypertrophy in hypertensive heart disease.^34,36^ In cluster 3, outcomes were generally less influenced by LV structures and more driven by LA dysfunction, reinforcing a metabolic-inflammatory route to atrial remodeling, which conventional CMR metrics may not fully capture.

Viewed across the spectrum of hypertensive heart disease, these 3 phenotypes represent distinct patterns of cardiac and vascular involvement, shaped by the interplay of elevated afterload, metabolic factors, and comorbidities. Cluster 1 represents a milder form with minimal cardiovascular remodeling and a more favorable prognosis. Cluster 3 represents an intermediate-risk phenotype dominated by MetS-driven diastolic dysfunction and moderate cardiovascular risk, especially for AF. Cluster 2 reflects a more advanced stage, where severe LV remodeling and vascular dysfunction converge, approaching the HF phenotype and carrying the highest risk of adverse outcomes. These phenotypes represent distinct, rather than strictly progressive, profiles that may potentially inform tailored therapeutic strategies.

### Comparison With Previous Clustering Studies

Several studies have applied clustering in hypertension populations, identifying clinically relevant phenotypes. Yang et al,^5^ using hierarchical clustering on the SPRINT cohort (n=9361), identified 4 groups, including an extra-risky subset resembling cluster 2, and an obese subset similar to cluster 3, though exclusion of diabetics limits comparability. Guo et al^6^ applied K-means to patients with hypertension (n=513), finding groups approximating cluster 3 (female, diabetic) and cluster 2 (high coronary artery disease prevalence), but without linking phenotypes to incident events.

Vaura et al^7^ (N=3726; FINRISK [National FINRISK Study] cohorts) and Bala et al^8^ (N=698; population-based study) described high-risk metabolic profiles in both sexes, confirming synergistic effects of metabolic dysfunction and hypertension on cardiovascular risk. Unlike these studies, which identified a single high-risk metabolic profile, our work revealed 2 distinct pathways: atherosclerosis-driven (cluster 2) and metabolic-driven (cluster 3). This greater resolution likely reflects the larger sample and broader variable set.

Katz et al^9^ (N=1273, Hypertension Genetic Epidemiology Network [HyperGEN]) integrated echocardiography with clinical metrics, identifying 2 distinct cardiovascular phenotypes of hypertension, including a high-risk group resembling cluster 2 with heart failure with preserved ejection fraction features. However, they did not relate phenotypes to incident events or identify an intermediate cardiovascular phenotype like cluster 3.

Despite differences in cohorts, variables, and algorithms, these studies broadly align with our findings, emphasizing that atherosclerotic burden and metabolic disorders drive cardiovascular risk beyond blood pressure alone.

### Clinical Implications

Identifying distinct hypertension phenotypes may help inform tailored prevention and management strategies, although evidence of improved outcomes compared with traditional care remains needed. For individuals resembling cluster 1, interventions should focus on promoting a healthy lifestyle and achieving adequate blood pressure control to prevent disease progression. In contrast, those mirroring cluster 3, with lower SBP but high metabolic and inflammatory burdens, require interventions beyond standard blood pressure targets. This involves addressing obesity, insulin resistance, and dyslipidemia, alongside medications aimed at improving diastolic dysfunction to reduce the risk of AF.^23^ Subjects resembling Cluster 2, instead, characterized by advanced atherosclerosis and target organ disease, require a more intensive approach, including strict blood pressure (including diastolic blood pressure) and lipid control, medications to counteract LV remodeling and improve microvasculature, and targeted lifestyle modifications to lower cardiovascular risk.

AF prevention should align with each phenotype’s distinct pathophysiological drivers. In cluster 2, limiting HF progression and LA remodeling is key to reducing the arrhythmogenic substrate. Conversely, in cluster 3, intensive metabolic and inflammatory control is paramount, as evidence suggests optimal metabolic management significantly lowers AF incidence in hypertension.^23^

### Strengths and Limitations

A key strength of this study is its large sample size and the extensive clinical data used for clustering. Unlike most prior clustering studies, which have explored cluster relevance through either clinical outcomes or imaging findings alone, this study integrates both, offering deeper insights into hypertensive heart disease risk pathways.

This study does not aim to redefine hypertension classification, as the identified clusters may vary depending on population characteristics and data availability. Rather, it highlights the potential of a multidimensional, data-driven approach to uncover clinically relevant phenogroups that could inform tailored interventions. Nevertheless, these findings align with other clustering studies in individuals with hypertension, supporting their broader clinical relevance. To move beyond proof-of-concept, however, external validation in independent data sets and prospective comparisons with traditional risk tools are necessary to confirm their added clinical utility.

The mediation findings highlight potentially important imaging biomarkers linking clinical phenotypes to outcomes; however, these should not be interpreted as proof of causality. Although they suggest mechanistic hypotheses with possible therapeutic relevance, prospective interventional studies are needed to validate these pathways. The CMR traits were selected as mediators based on their recognized role as markers of hypertensive target-organ damage along the causal pathway to cardiovascular events, making them biologically and clinically plausible. However, other potential mediators, such as circulating biomarkers, ECG traits, or vascular parameters, were not explored and warrant future investigation.

Several limitations related to the data set used should be noted. The cohort is predominantly middle-aged and White, with a near-exclusion of non-White individuals, which may limit generalizability to more diverse populations. Although the overall sex distribution was balanced, the clustering process identified phenotypes that were sex-skewed, with cluster 1 and 3 predominantly female and CLUSTER 2 predominantly male. This may influence the interpretation of risk profiles and generalizability across sexes. Self-reported data (eg, lifestyle, conditions) may be biased, leading to some inaccuracy. Although most clustering inputs were collected at the imaging visit, laboratory biomarkers were obtained years earlier at baseline. This temporal gap may introduce bias beyond misclassification, including survivorship and treatment effects between visits. However, our sensitivity analysis excluding participants with long baseline-CMR intervals showed consistent cluster structures and associations, suggesting these factors had a limited impact on the main findings. Detailed information on medication classes, dosages, and durations (including antihypertensive, lipid-lowering, and other relevant therapies) was not available for this work and may have influenced the observed phenotypic patterns. Genetic data from the UK Biobank were not integrated, which could have enhanced risk stratification and will be explored in future work. Future work will aim to integrate both genetic and medication data to improve clustering precision and assess whether phenotype-guided strategies can support more tailored interventions and improve long-term cardiovascular outcomes.

Despite greater remodeling and presumed fibrosis, clusters 2 and 3 exhibited lower native T1 values than cluster 1, aligning with prior UK Biobank findings yet conflicting with other studies.^16,37^ Variations in acquisition methods or complex fibrotic patterns within hypertension, which perhaps native T1 alone cannot fully capture, may account for these discrepancies.^38,39^ Incorporating contrast-enhanced imaging and extracellular volume quantification, which are unavailable in this data set, could enhance the assessment of fibrotic patterns in hypertension and clarify such discrepancies.^39^

Finally, although posthoc analyses and bootstrapping supported the clinical significance and confirmed stability of the clustering method, replicating these findings in independent cohorts is essential to confirm their generalizability.

### Conclusions

Unsupervised clustering of clinically available data in a large UK Biobank cohort of hypertension subjects identified 3 distinct phenotypes with differing risk profiles, cardiovascular imaging characteristics, and outcomes. These phenotypes likely reflect different underlying mechanisms and support a more individualized approach to risk assessment. These findings underscore the heterogeneity of hypertensive heart disease and demonstrate the potential of data-driven strategies to complement traditional risk stratification and guide personalized care.

### Perspectives

This study highlights the potential of unsupervised clustering to refine risk stratification in hypertension. Using diverse, patient-specific data from a large UK Biobank cohort, we identified phenotypes with distinct risk profiles and cardiovascular trajectories, revealing heterogeneity often missed by traditional approaches. Compared with previous clustering studies limited by smaller samples or narrower variable sets, this work offers a more comprehensive view of hypertensive heart disease. The differential role of imaging features across clusters suggests diverse mechanisms driving risk, supporting the idea that hypertension is not a uniform condition and may benefit from phenotype-based management. Future research should validate these findings in independent cohorts and assess whether phenotype-guided strategies improve outcomes. Although observational and hypothesis-generating, this study provides a foundation for testing the clinical validity of phenotype-based approaches and supports a shift toward more personalized hypertension management.

## Article Information

### Acknowledgments

Figure 6 in Supplemental Methods was generated with Biorender.

### Author Contributions

E. Rauseo conceived the study, performed analyses, and wrote the article. G.G. Slabaugh, N. Aung, and S.E. Petersen supervised the work. A.M. Salih, M. Abdulkareem, and C.R.S. Banerji advised on machine learning, and J. Cooper on statistics. All the authors contributed to and approved the final article.

### Sources of Funding

This work was supported by the NIHR Barts Biomedical Research center (NIHR203330), a partnership of Barts Health NHS Trust, Queen Mary University of London, St George’s University Hospitals NHS Foundation Trust, and St George’s University of London. Barts Charity (G-002346) funded UK Biobank data access. S.E. Petersen acknowledges the British Heart Foundation for funding manual analysis to establish a CMR reference standard in 5000 UK Biobank scans (www.bhf.org.uk; PG/14/89/31194).

### Disclosures

S.E. Petersen provides consultancy to Circle Cardiovascular Imaging Inc, Calgary, Alberta, Canada. G.G. Slabaugh serves on the Scientific Advisory Board to BioAI Health, Boston. The other authors report no conflicts.

### Supplemental Material

Supplemental Methods

Supplemental Results

Tables S1–S12

Figures S1–S10

References 40–53

## Supplementary Material
