# Supplementary material for: Clinical Phenotypes in Hypertension: A Data-Driven Approach to Risk Stratification
Source: Hypertension. 2025 Dec 11;83(6):e25187. doi: 10.1161/HYPERTENSIONAHA.125.25187 (PMC13189382; doi:10.1161/HYPERTENSIONAHA.125.25187)
Supplement: Supplementary file 1 [file hyp-83-e25187-s001.docx]

**Supplemental Materials**

**Clinical phenotypes in hypertension: a data-driven approach to risk stratification**

Elisa Rauseo ^1,2.3*^, Ahmed M. Salih ^1,2,4,5^, Jackie Cooper ^1^, Musa Abdulkareem ^1,2^, Christopher R.S. Banerji ^6,7,8^, Sucharitha Chadalavada ^1,2^, Hafiz Naderi ^1,2^, Patricia B Munroe ^1^, Anthony Mathur ^2,9,10^, Nay Aung ^1,2^, Gregory G. Slabaugh ^3,11^, Steffen E. Petersen ^1,2^

*^1^ William Harvey Research Institute, NIHR Barts Biomedical Research Centre, Queen Mary University London, Charterhouse Square, London, EC1M 6BQ, UK*

*^2^ Barts Heart Centre, St Bartholomew’s Hospital, Barts Health NHS Trust, West Smithfield, EC1A 7BE, London, UK*

*^3^ Digital Environment Research Institute, Queen Mary University of London, United Kingdom*

*^4^ Department of Population Health Sciences, University of Leicester, Leicester UK*

*^5^ PRIME Lab, Scientific Research Center, University of Zakho, Kurdistan Region, Iraq*

*^6^ The Alan Turing Institute, London, UK*

*^7^ King’s Comprehensive Cancer Centre, King’s College London, London UK*

*^8^ University College London NHS Trust, London UK*

*^9^ Centre for Cardiovascular Medicine and Devices, William Harvey Research Institute, Queen Mary University of London, London, UK*

*^10^ NIHR Barts Biomedical Research Centre, Queen Mary University of London, Charterhouse Square, London, EC1M 6BQ*

*^11^ School of Electronic Eng. & Computer Science, Queen Mary University of London, United Kingdom*

**Corresponding author**

Dr Elisa Rauseo

Contacts: e.rauseo@qmul.ac.uk

William Harvey Research Institute, Queen Mary University London

Charterhouse Square, London, EC1M 6BQ, UK

1. **Supplemental Methods 1 – Imaging parameters.**
2. **Supplemental Methods 2 – Analysis workflow.**
3. **Supplemental Results – Sensitivity analyses.**
4. **Supplemental References.**
5. **Table S1. Inclusion and exclusion criteria for defining the hypertensive study cohort.**
6. **Table S2. Phenotypic features scrutinised for clustering, with corresponding UK Biobank data fields.**
7. **Table S3. UK Biobank data sources used to identify clinical outcomes.**
8. **Table S4. Baseline clinical characteristics of the study cohort.**
9. **Table S5. Health-related lifestyle behaviours according to the identified clusters.**
10. **Table S6. Laboratory and electrocardiographic characteristics stratified by clusters.**
11. **Table S7. Association of clusters with adverse outcomes on Cox proportional hazards analysis – sensitivity analysis (cases with less than 6 months follow-up have been excluded).**
12. **Table S8. Summary of Cox model performance*s*.**
13. **Table S9. Associations of CMR metrics with clustering.**
14. **Table S10. Association of clusters with adverse outcomes on Cox proportional hazards analysis – sensitivity analysis (cardiomyopathy cases are excluded).**
15. **Table S11. Main clinical characteristics stratified by cluster - sensitivity sample after excluding participants with long intervals between baseline and CMR assessment (temporal heterogeneity).**
16. **Table S12. Association of clusters with adverse outcomes on Cox proportional hazards analysis -sensitivity analysis (temporal heterogeneity).**
17. **Figure S1. Kaplan-Meier survival analysis for incident events stratified by clusters.**
18. **Figure S2. Associations between CMR metrics and clustering - sensitivity analysis (prevalent conditions are excluded).**
19. **Figure S3. Associations between CMR metrics and clustering - sensitivity analysis (cardiomyopathy cases are excluded).**
20. **Figure S4. Proportion of MACE risk mediated by CMR features across clusters - sensitivity analysis (cardiomyopathy cases are excluded).**
21. **Figure S5. Proportion of death risk mediated by CMR features across clusters - sensitivity analysis (cardiomyopathy cases are excluded).**
22. **Figure S6. Elbow method for identifying the optimal number of clusters: FAMD + K-means approach -sensitivity analysis (temporal heterogeneity).**
23. **Figure S7. Radar charts showing the top 32 features contributing to clustering - sensitivity analysis (temporal heterogeneity).**
24. **Figure S8. Associations between CMR metrics and clustering - sensitivity analysis (temporal heterogeneity).**
25. **Figure S9. Proportion of MACE risk mediated by CMR features across clusters - sensitivity analysis (temporal heterogeneity).**
26. **Figure S10. Proportion of AF risk mediated by CMR features across clusters - sensitivity analysis (temporal heterogeneity).**

**Supplemental Methods 1 - Imaging parameters.**

The acquisition protocol of CMR imaging in UK Biobank has been described in detail by Petersen et al. ^1^ The CMR images were acquired with a 1.5 Tesla scanner (MAGNETOM Aera, Syngo Platform VD13A, Siemens Healthcare, Erlangen, Germany) with 48 receiver channels, an 18-channel anterior body surface coil used in combination with 12 elements of an integrated spine coil, enabling 32-channel imaging and ECG gating for cardiac synchronisation. The CMR scans were performed in dedicated UK Biobank imaging centres using uniform staff training and equipment, according to the above protocol. The cardiac assessment included a combination of long-axis (LAX) cines (horizontal long-axis – HLA, vertical long-axis – VLA, and left ventricular outflow tract –LVOT cines, both sagittal and coronal) and a complete short-axis (SAX) stack covering both left and right ventricles (LV, RV) acquired using balanced steady-state free precession sequences. All cine images were acquired at one slice per breath-hold.

CMR image segmentation was performed manually in the initial ~5,000 UK Biobank studies by two image-analysis core laboratories using CVI42 version 5.1.1 (Circle Cardiovascular Imaging Inc, Calgary, Canada) as previously described. ^2^ This expert-annotated dataset was then used to develop an automated image analysis pipeline with integrated quality control processes, validated in a large sample of over 30,000 UK Biobank CMR studies. ^3^ Using this automated image analysis pipeline, conventional CMR indices of cardiac function and structure (LV and RV volumes, stroke volumes and ejection fraction; LV mass) were extracted. ^3^ Using this automated segmentation, the LV maximum wall thickness was defined as the greatest epicardial-to-endocardial distance across the 16 American Heart Association segments at end-diastole. Additional cardiac indices able to capture changes in myocardial function in relation to structural chamber remodelling were also derived, including LV mass-to-volume (M/V) ratio and LV global function index (LVGFI). The M/V ratio was calculated as LV mass divided by LV end-diastolic volume. Increased M/V ratio has been linked with cardiac remodelling and poorer cardiovascular outcomes. LVGFI is a validated measure of LV cardiac performance that integrates structural components of adverse myocardial remodelling into LV function assessment, for which the formula is described elsewhere. ^4^ Higher LVGFI indicates better LV cardiac performance and provides incremental value over LV ejection fraction in predicting adverse cardiovascular events.

The left atrial (LA) volumes and functional indices were derived using a fully automated 3D segmentation pipeline previously validated on a UK Biobank cohort. ^5^ This method accurately reconstructed LA geometry from CMR images using a statistical shape model. The analysis extracted key LA volumes, including maximum, minimum, and pre-atrial contraction volumes, to compute functional indices such as total, passive, and active emptying fractions (LAEF), based on formulae described elsewhere. ^6,7^ The LA expansion index, a marker of LV reservoir function, was also assessed using the formula: ([LA volume max−LA volume min]/LA volume min) * 100%. ^7^ Quality control involved removing statistical outliers (three times the interquartile range) to ensure accuracy, aligning with prior UK Biobank CMR studies. ^2^

Myocardial native T1 mapping, a quantitative technique for tissue characterisation, was assessed using a single mid-ventricular SAX slice acquired with the Shortened Modified Look-Locker Inversion Recovery (ShMOLLI) sequence. Global native T1 values were extracted using a fully automated, quality-controlled tool. ^8^ Studies with a predicted Dice score below 0.7 were excluded to ensure data reliability.

Global longitudinal strain (GLS), global circumferential strain (GCS), global radial strain (GRS), and torsion for both LV and RV were derived from CMR feature tracking (FT) using automated batch processing (CVI42 prototype 5.13.7) as described in previous publications. ^9,10^ The analysis was performed on 45,700 UK Biobank scans, with automated contours generated for SAX and LAX cine images, defining LV end-diastole and end-systole. Temporal smoothing assisted tracking, with LV diastole as the reference phase. For this study, peak GLS, GCS, GRS, and torsion were selected, with peak strain calculated as the average of all myocardial points with tracked deformation. Quality control followed a validated statistical outlier removal method used in previous UK Biobank studies. ^9,10^ Outliers were excluded based on values exceeding three times the interquartile range (IQR) and nonsensical values (e.g., GLS/GCS > 0, GRS < 0, indicating software errors). This method was validated in a subset of 1,957 scans, showing over 97% agreement with visual expert assessment, confirming its reliability for large datasets. ^9,10^

Aortic distensibility (AoD), total arterial compliance (TAC), and systemic vascular resistance (SVR) derived from CMR were considered descriptors of arterial health. AoD is a measure of local aortic compliance and is considered the major contributor to arterial stiffness. TAC represents a measure of global arterial stiffness reflecting the effect of both large and small arteries’ properties; SVR, instead, represents the resistive component of the LV afterload, primarily determined by the small vessel tone and depending on microvascular properties. AoD was estimated by measuring the relative change in the area of the thoracic aorta on CMR images divided by central pulse pressure using Vicorder^®^ readings at the time of scanning (<https://biobank.ndph.ox.ac.uk/showcase/ukb/docs/vicorder_explan_doc.pdf>). Aortic distensibility values were obtained from a previous analysis of a large subset of the UK Biobank imaging studies using a fully automated image analysis pipeline embedded with purpose-designed quality control. ^11^ CMR values of cardiac output and stroke volume were used to estimate TAC (LV stroke volume/central pulse pressure) and SVR (mean arterial pulse pressure/cardiac output). As the arterial load highly depends on body size, we indexed TAC and SVR for body surface area (BSA).

**Supplemental methods 2 - Analysis workflow.**

The overall analysis comprised three main stages outlined below: (1) data preparation, (2) clustering analysis, and (3) *post hoc* statistical analyses.

**1)** ***Data preparation***

From the 79 clinical features scrutinised as potential input for clustering (Table S2), those with more than 20% missingness (microalbumin in urine) were excluded. As the cohort was mainly composed of Caucasian individuals (97%), ethnicity was also excluded as an input variable for clustering. This left 77 clinical features for clustering. Among these, the remaining missing values were imputed based on data type. Specifically, missing values were imputed using simple, type-specific imputation: the mean (or median, where distributions were skewed) for continuous variables and the most frequent category for categorical variables. This deterministic approach was chosen to preserve the original data structure and avoid introducing artificial variance, which is particularly important in unsupervised clustering where outcome information is not available. The cleaned dataset was then utilised as input for the clustering analysis. No explicit outlier removal step was performed. Continuous variables were standardised before applying FAMD, which minimises the influence of individual extreme values by projecting data onto components that capture shared variance across participants.

**2) *Clustering analysis***

Three clustering approaches were evaluated to identify the method that best balanced clustering performance, computational efficiency, and clinical relevance. While this paper focuses on the results from approach 2, which was selected for its superior performance and interpretability, we also provide an overview of approaches 1 and 3 to illustrate the rationale behind our final choice.

1. Approach 1 - K-Prototypes Clustering:

As the dataset included both continuous and categorical variables, we initially applied the K-prototypes algorithm (*modes.KPrototypes* function from the *kmodes* package (0.12.2) implemented in Python) to identify phenotypic clusters. This was performed on two versions of the dataset: one including all variables (n =77) and a reduced version (n=65) obtained by excluding highly correlated features using the correlation matrix (thresholds for exclusion: Pearson correlation > 0.8 for continuous; Cramer’s V > 0.8 for categorical variables). Clustering performance was evaluated for cluster (*k*) values ranging from 1 to 10 using the elbow method, which plots the total clustering cost (a unitless measure reflecting the sum of within-cluster dissimilarities) against the number of clusters, or *k*. A clear elbow point, indicating diminishing returns with increasing *k* was observed at *k*=3 (cost = 4 x10^15^). This was further confirmed using the *KneeLocator* function from the *kneed* package (0.8.1) implemented in Python. Both the full and reduced variable sets produced consistent results, supporting a three-cluster solution. However, the considerable computational demands of K-prototypes in this high-dimensional setting (multi-day runtimes on GPU) limited its practicality for further refinement. Therefore, alternative approaches were explored to enable more efficient and interpretable clustering.

1. Approach 2 - Dimensionality reduction + K-means Clustering:

To improve computational efficiency and reduce the complexity of the dataset, we first applied Factorial Analysis of Mixed Data (FAMD), a generalisation of Principal Component Analysis (PCA) tailored for mixed datasets (containing both continuous and categorical variables), executed with the *Prince* library for multivariate exploratory data analysis in Python. The continuous variables were standardised, and categorical variables were one-hot encoded and scaled appropriately. The resulting matrix was then subjected to PCA to obtain principal components (PCs). We selected 40 PCs that explained more than 80% of dataset variance (Figure 1). These components were then used as input for clustering using the K-means algorithm (*sklearn.cluster.KMeans* module, from the scikit-learn library, version 1.3.2).

To determine the optimal number of clusters (*k*), we applied the elbow method, which evaluates clustering performance based on inertia, defined as the within-cluster sum of squared distances. A clear elbow point was observed at *k* = 3 (inertia = 135,000), indicating an optimal balance between cluster compactness and model simplicity (Figure 2).

In addition, we assessed clustering quality using the Silhouette score, which measures how similar each point is to its own cluster compared to other clusters, ranging from -1 (poor separation) to +1 (well-separated). The scores were 0.097 for *k* = 2 and 0.087 for *k* = 3, both reflecting modest but acceptable separation in a complex, high-dimensional dataset. Although the silhouette score was slightly higher for *k* = 2, the elbow method and clinical interpretability supported the use of a three-cluster solution.

1. Approach 3 - Dimensionality reduction + K-means Clustering (reduced feature set):

To further explore potential improvements, we repeated the dimensionality reduction and clustering pipeline (FAMD + K-means) using the reduced dataset of 65 variables, in which highly correlated features had been removed prior to analysis.

While this approach produced clustering results similar to those from the full dataset (approach 2), performance was slightly worse. Specifically, the Silhouette scores were lower (0.078 for *k* = 2 and 0.072 for *k* = 3), and inertia was higher (elbow was identified at *k* = 3: 152,000), suggesting reduced cluster compactness and separation. These findings indicate that dimensionality reduction, rather than prior feature elimination, was the key factor driving improved clustering performance.

Given the high computational burden of K-Prototypes (approach 1) and the lower performance of clustering after removing correlated features (approach 3), approach 2 (FAMD + K-Means) was selected for final analysis. A three-cluster solution (*k* = 3) was chosen based on the elbow method, which consistently indicated this as optimal across all approaches, and a Silhouette score comparable to that of *k* = 2 (the highest). Importantly, the three-cluster model revealed more granular and clinically interpretable phenotypes than the two-cluster alternative (Figure 3). Of note, one cluster appeared elongated in the two-dimensional FAMD projection (Figure 3). This shape may reflect correlations among continuous features that form a gradient within the same phenotype, rather than poor separation or instability of the K-Means algorithm. As clustering was performed on the full multidimensional space, such elongation in two-dimensional projections does not affect the validity or interpretability of the clustering structure. Table 1 below summarises the quantitative metrics for each clustering method, including the number of clusters tested, the estimated computational burden, and interpretability considerations that informed the final method selection.

After selecting the clustering methods and optimal k, clustering stability was assessed using bootstrapping. The procedure involved iteratively resampling 80% of the dataset across multiple iterations (n = 100) and reapplying K-Means clustering (k = 3) to each resampled subset. The K-Means algorithm used k-means++ initialization with 10 random starts (n_init = 10) and independent random seeds per iteration to minimize sensitivity to initial centroid placement. The Adjusted Rand Index (ARI), which measures the agreement between the original and resampled cluster assignments while correcting for chance, was used to quantify stability. A higher ARI (closer to 1) indicated that the clustering structure was robust and reproducible.

To enhance the interpretability of clustering and validate cluster assignments, we applied Shapley Additive Explanations (SHAP), an explainable AI technique that quantifies the contribution of individual features to model predictions. A supervised classifier was trained on the 40 FAMD-derived PCs to predict the unsupervised cluster labels. We tested both Random Forest and LightGBM algorithms using 80/20 train-split of the dataset. Both models achieved 98% accuracy, precision, recall, and F1 score on the test set, supporting the robustness and separability of the clusters identified through unsupervised learning. SHAP values were then computed to identify which PCs most strongly influenced cluster predictions. The analysis revealed that PC0 (37%) and PC1 (32%) were the most important components driving clustering (Figure 4). To interpret the PCs in clinical terms, we mapped these PCs back to their original input features using the *famd.columns_correlations()* function (Prince, Python). The top 32 contributing features were identified, primarily anthropometric and metabolic factors, along with ECG parameters. Sex was the only categorical feature with a significant role, while others contributed minimally (see Table 1 in the main manuscript). These SHAP-ranked features were used to describe phenotypic differences between clusters, improving clinical interpretability and ensuring that the data-driven clustering retained clinical relevance. This supervised modelling approach also served as a confirmatory step, reinforcing that the unsupervised clustering produced well-separated and clinically meaningful groups.

The clustering analysis presented in this paper did not include urinary microalbumin concentration (Field 30500), as the variable had more than 20% missing values and was therefore excluded during preprocessing. However, recognising the clinical relevance of microalbuminuria in cardiovascular risk assessment, especially in the context of HTN, and the fact that missingness due to values below the detection limit can be addressed through conservative imputation, as suggested by UK Biobank documentation, we conducted a sensitivity analysis. Values below the detection limit were imputed as 6.7 mg/L based on Field 30505, and remaining missing values were imputed using the median. The clustering workflow, including scaling, FAMD dimensionality reduction, and K-means clustering, was repeated with microalbuminuria included. SHAP analysis was then applied to assess feature importance in predicting cluster assignments. The same clustering structure and top contributing components emerged (Figure 5 below), with microalbuminuria not ranked among the top 32 influential features. These findings indicate that the inclusion of microalbuminuria did not materially influence the clustering results, supporting the robustness of the original model.

**Figure 1. Determining the Number of Principal Components in FAMD.**


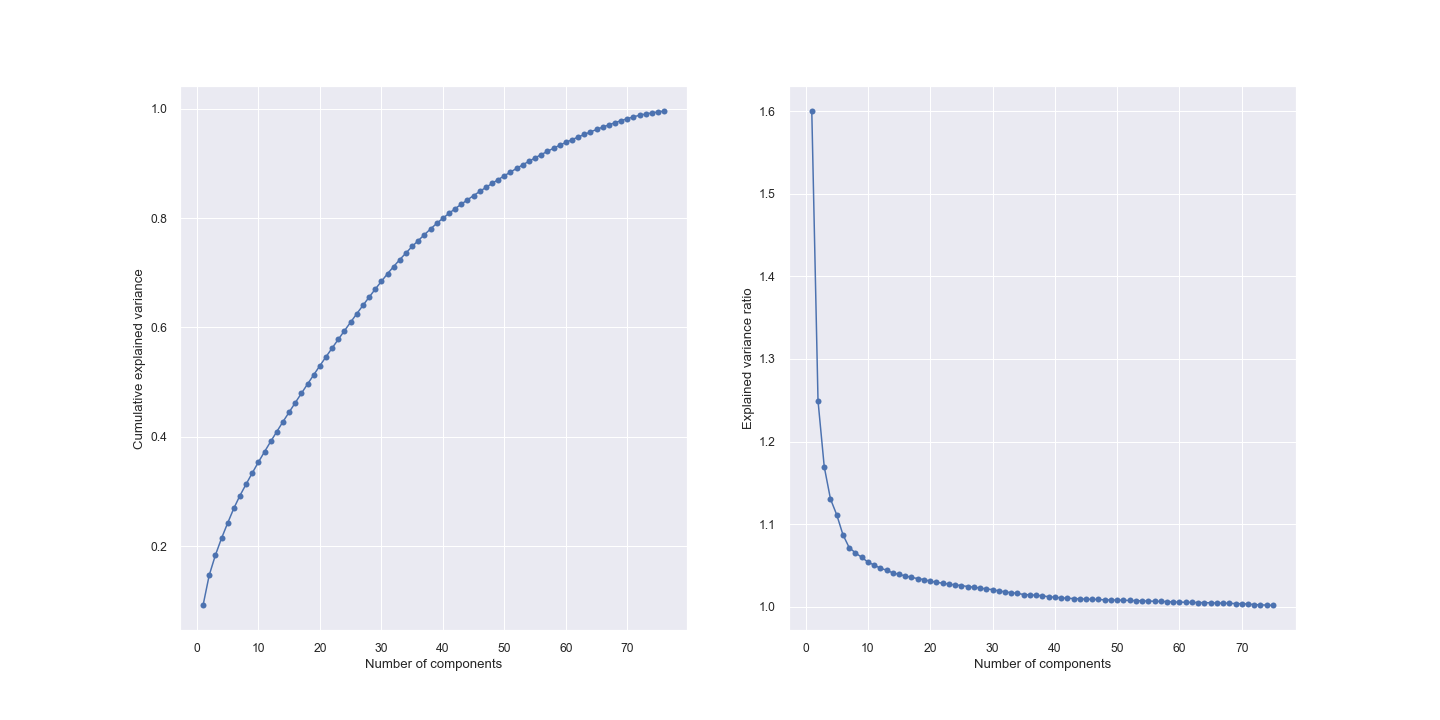


The left plot shows the cumulative explained variance, indicating that approximately 40 principal components (PCs) capture over 80% of the dataset’s variance. The right plot displays the explained variance ratio per component, with a marked decline after the initial components, supporting the selection of 40 PCs for clustering.

**Figure 2. Elbow method for identifying the optimal number of clusters: FAMD + K-means approach.**


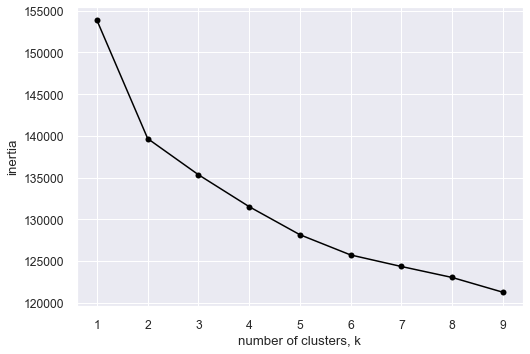


On the y-axis is the inertia for each value of clusters, k, (x-axis) n a given range (1-10). The optimal number of clusters that balances minimising the variance within clusters is identified as the point where an elbow-like bend with a lesser inertia value is observed. In this case, as confirmed by the knee locator, the elbow is at k=3, indicating an optimal number of clusters is three.

**Figure 3. Visualisation of Three-Cluster Solution Using K-Means.**


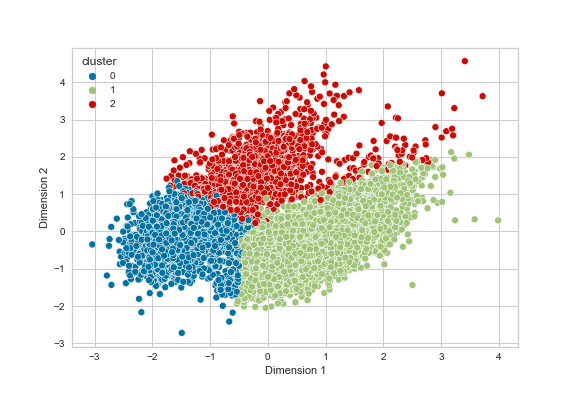


Scatter plot displaying the three clusters identified through K-means clustering, projected onto the first two principal components. Each point represents an individual, with colours denoting cluster membership. The evident separation of clusters indicates distinct phenotypic patterns within the hypertensive cohort.

**Table 1. Summary of quantitative metrics, estimated computational burden, and interpretability across clustering approaches and number of clusters (k) explored.**

| **Approach** | **Variable set** | **k** | **Cost/ Inertia** | **Silhouette Score** | **Elbow point** | **Estimated computational time** | **Interpretability** |
| --- | --- | --- | --- | --- | --- | --- | --- |
| K-Prototypes | 77 | 2 | ~6.8 × 10¹⁵ |  |  | ~ 2 days (GPU) | Initial run; high cost |
| K-Prototypes | 77 | 3 | ~4.0 × 10¹⁵ |  | k=3 | ~ 2 days (GPU) | Optimal balance; visible elbow |
| K-Prototypes | 77 | 4 | ~3.0 × 10¹⁵ |  |  | ~ 2 days (GPU) | Diminishing gain, more complex interpretation |
| K-Prototypes | 65 | 2 | ~790,000 |  |  | ~several hours (CPU) | High cost, simple model |
| K-Prototypes | 65 | 3 | ~755,000 |  | k=3 | ~several hours (CPU) | Best trade-off (clear elbow); interpretable and cost-efficient |
| K-Prototypes | 65 | 4 | ~730,000 |  |  | ~several hours (CPU) | Marginal improvement, reduced interpretability |
| FAMD + KMeans | 77 | 2 | ~140,000 | 0.097 |  | ~2 minutes (CPU) | Best silhouette, but limited granularity |
| FAMD + KMeans | 77 | 3 | ~135,000 | 0.087 | k=3 | ~2 minutes (CPU) | Slightly lower silhouette, better interpretability and elbow match |
| FAMD + KMeans | 77 | 4 | ~131,000 | 0.065 |  | ~2 minutes (CPU) | Lower silhouette, marginal gain in compactness, limited interpretability |
| FAMD + KMeans | 65 | 2 | ~156,000 | 0.078 |  | ~2 minutes (CPU) | Similar pattern to full model but lower compactness |
| FAMD + KMeans | 65 | 3 | ~152,000 | 0.072 | k=3 | ~2 minutes (CPU) | Acceptable compromise: elbow preserved despite slightly worse metrics |
| FAMD + KMeans | 65 | 4 | ~146,000 | 0.055 |  | ~2 minutes (CPU) | Decreasing Silhouette, marginal gain in inertia |

**Inertia / Cost:** In K-Means, inertia reflects the within-cluster sum of squared distances, with lower values indicating more compact clusters. In K-Prototypes, the analogous term is "cost", which integrates both numerical distances and categorical mismatches. These metrics are not directly comparable across methods.

**Silhouette Score:** This metric indicates how well each observation fits within its cluster versus others. The values range from -1 (misclassified) to +1 (well-clustered). This metric is not applicable to K-Prototypes due to its handling of mixed data types.

**Elbow Point:** The elbow marks the value of k beyond which reductions in cost/inertia become negligible. It is determined visually from the cost/inertia plot. In our analysis, k = 3 consistently emerged as the most balanced choice.

**Estimated computational time:** Approximate runtime estimates are provided, but they may vary depending on computational resources. K-Prototypes required a GPU for the full 77-variable set (approx. 2 days), while FAMD + K-Means completed within minutes on a standard CPU.

**Interpretability:** The choice of the optimal approach and number of clusters (k) was guided by a combination of performance metrics (inertia/cost, silhouette score), interpretability, and computational feasibility. Increasing k generally improves compactness but reduces interpretability. While k=2 showed higher silhouette scores in some cases, it offered less granularity and reduced clinical usefulness. Across all methods, k=3 consistently aligned with elbow points and produced more interpretable phenotypes. FAMD + K-Means with 77 variables was ultimately selected due to its balance of compactness, interpretability, and computational efficiency, particularly compared to the much more resource-intensive K-Prototypes algorithm.

**Figure 4. List of most informative feature predictors (PCs) based on mean SHAP value.**


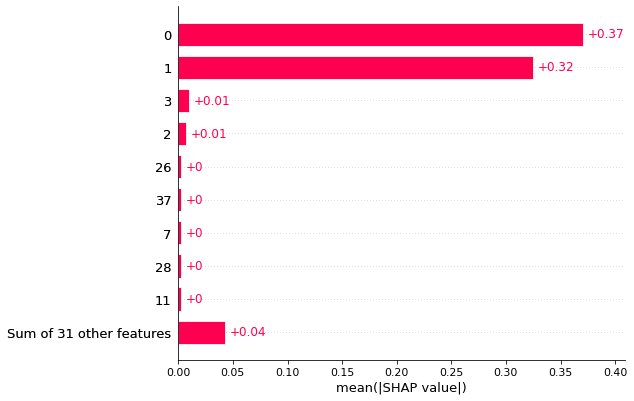


**Figure 5. SHAP feature importance plot for principal components after inclusion of urinary microalbumin.**


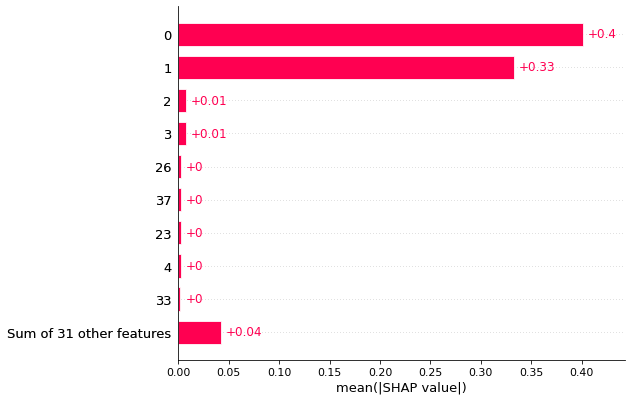


**3) *Post hoc analyses***

Clustering was performed independently of clinical outcomes and CMR metrics, which were subsequently used in *post hoc* analyses to assess the clinical relevance of the identified phenotypes and better characterise underlying risk profiles. These analyses examined associations between cluster membership and clinical outcomes, cardiac structure/function metrics, and the potential mediating role of cardiovascular imaging features in outcome risk.

*Clustering and clinical outcomes*

To evaluate associations between cluster membership and clinical outcomes, we developed Cox proportional hazards models for each outcome of interest, using the cluster with the lowest risk profile as the reference group. A cause-specific Cox model was used in all cases, as this is the recommended approach for estimating causal associations in the presence of competing risks. ^12,13^ The proportional hazards assumption was assessed for all Cox models using Schoenfeld residuals (lifelines package, Python). No significant violations were detected, and graphical inspection confirmed that the assumption of proportionality was adequately met.

To ensure model validity and adherence to the at-risk assumption, we excluded individuals with a history of the specific condition being modelled at baseline. For example, participants with prevalent AF were excluded from the AF model, while those with previous MI, stroke, or PAD were excluded from the atherosclerotic outcome model. Similarly, individuals with prior cardiovascular diseases (including both atherosclerotic events and AF) were excluded from the MACE model. Of note, the HF model did not require excluding any prevalent HF cases, as these were already excluded by the study cohort by design of this study. For all-cause mortality and non-cardiovascular mortality, no exclusions were made based on baseline conditions, as these were not disease-specific endpoints.

To account for baseline differences in cardiovascular risk and address the potential confounding introduced by pre-existing disease, models were adjusted for diabetes, hypercholesterolaemia, as well as for a composite variable indicating any prior CVDs, defined as a history of MI, stroke, PAD, or AF. Heart failure was not included in this composite since prevalent HF cases were already excluded from the cohort. These specific conditions were selected because they differed across clusters and could influence both subsequent outcomes and CMR findings. Including them as a composite variable provided a parsimonious method of adjustment, avoiding overfitting while adequately addressing confounding related to prior disease burden. For outcomes where all individuals with prior events had been excluded (e.g., MACE), the composite covariate was not used. Other traditional cardiovascular risk factors such as age, BMI, smoking, alcohol use, and socioeconomic status were already included as inputs in the clustering process, and were not reintroduced in *post hoc* models. Re-adjusting for these variables would risk overcorrecting for characteristics already embedded in the definition of each cluster, potentially obscuring meaningful differences in outcomes attributable to the clustered phenotypes.

To further explore potential competing risks, we included an additional Cox model for non-cardiovascular death. This allowed us to evaluate whether non-CVD mortality could preclude the occurrence of cardiovascular events. Although the absolute number of competing events was low (<2%) and consistently lower than the number of events for each primary outcome, this model reinforced our interpretation of the primary cause-specific models.

For all Cox models, hazard ratios (HR) were estimated, and Kaplan-Meier survival analyses with log-rank tests were used to compare event-free survival across clusters. The Kaplan-Meier method can lead to bias in the estimation of absolute risks when competing risks are present. However, as the percentage of participants with competing events was low (<2%) and lower than the percentage experiencing each outcome of interest, there is unlikely to be substantial bias in the Kaplan-Meier estimates. ^14^

As a sensitivity analysis, we repeated all Cox models after excluding individuals with less than six months of follow-up, to minimise potential reverse causality from subclinical disease present at baseline.

To evaluate model performance and quantify the added prognostic value of clustering beyond established cardiovascular risk factors, we calculated Nagelkerke’s pseudo R² (as a measure of explained variance) and the concordance index (C-statistic) to assess discrimination. These metrics were reported for three model types: (i) unadjusted models including only cluster variables, (ii) models including only clinical covariates (i.e., prevalent cardiovascular conditions), and (iii) fully adjusted models including both clusters and covariates. Pseudo R² provides insight into the proportion of variation explained by each model, although absolute values are typically low in survival analyses. Since pseudo R² is most interpretable when used for within-sample model comparisons, we ensured that all model types applied the same exclusion criteria (i.e., removal of prevalent cases specific to each outcome). The concordance index further characterised model discrimination and allowed us to evaluate the incremental predictive value of clustering by comparing fully adjusted models against covariate-only models.

*Clustering and CMR metrics*

To explore the association between clustering and cardiac structural or functional parameters, we performed multivariable linear regression models linking cluster membership to CMR metrics. These models were adjusted for the same covariates used in the Cox models. To ensure that the associations between CMR parameters and clusters did not reflect sequelae of existing disease rather than distinct hypertensive phenotypes, we performed a sensitivity analysis excluding participants with any prior cardiovascular condition (AF, MI, PAD, or stroke). The CMR-cluster associations were then repeated in this restricted sample, adjusting for the remaining major covariates (diabetes and high cholesterol), consistent with the approach used for MACE in the Cox models.

To investigate whether specific CMR features mediated the associations between clusters and clinical outcomes, we conducted mediation analyses using logistic regression models (PROCESS, R & SPSS). ^15^ CMR traits were selected as candidate mediators based on their established role as markers of hypertensive target-organ damage and their recognised position on the causal pathway between elevated blood pressure and cardiovascular events. These traits represent downstream effects of prolonged hypertension and intermediate steps toward adverse outcomes. We hypothesised that their varying levels across clusters could help explain differences in outcome risk, reflecting heterogeneous contributions to cardiovascular burden. Each cluster was treated as a categorical exposure, with binary clinical outcomes as the dependent variables, and individual CMR metrics as continuous mediators. Each CMR metric was tested in a separate univariable mediation model. For consistency, we applied the same exclusion criteria and covariate adjustment strategy as in the Cox models. Total, direct, and indirect effects were estimated in the log-odd scale, then exponentiated to obtain odds ratios (ORs) with 95% confidence intervals. The proportion mediated by each CMR feature was calculated as the ratio of the indirect effect (through the CMR feature) to the total effect, after converting to ORs, using a published method. ^16^ This reflects the extent to which the association between cluster membership and each clinical outcome is explained by a given CMR metric, after adjusting for confounders. For instance, a mediation proportion of 67% indicates that 67% of the observed effect is attributable to the pathway through the CMR feature (e.g., LV mass), while 33% reflects a residual direct association. Since each mediation model was fitted independently, proportions mediated are not additive across CMR metrics. A conceptual illustration of the mediation framework (paths a, b, c, and c′) is shown in Figure 6 below.

**Figure 6. Mediation pathways between clustering, CMR metrics, and clinical outcomes.**

Path **a** represents the association between cluster membership and the CMR metric (mediator); path **b** represents the association between the CMR metric and the clinical outcome, adjusted for cluster; path **c′** is the direct effect of cluster on the outcome after accounting for the mediator; and path **c** is the total effect of cluster on the outcome.
The product of paths **a** and **b** quantifies the indirect (mediated) effect, and the total effect is defined as **c** *=* **c′ + (a × b)**.
The proportion mediated is calculated as *(a × b) / c*, representing the percentage of the total effect explained by the indirect pathway. This figure is generated with Biorender.

*Sensitivity analysis 1 (cardiomyopathy cases excluded)*

Since the HTN cohort included some individuals with early-stage cardiomyopathy (n = 1,513, including both ischaemic and non-ischaemic forms), we repeated all post hoc analyses after excluding these participants. This allowed us to determine whether the identified clusters reflected hypertensive phenotypes rather than being influenced by pre-existing myocardial disease.

*Sensitivity analysis 2 (temporal heterogeneity)*

To ensure that the identified clusters and their associations were not influenced by temporal heterogeneity between baseline and CMR assessments, we performed an additional sensitivity analysis. This analysis aimed to verify that the observed phenotypic structure reflected genuine differences in participant characteristics rather than artefacts introduced by the time gap between data collections.

We quantified the interval between baseline and imaging visits for all participants (median = 112.4 months, IQR = 94.2–125.0 months; skewness = - 0.43) and excluded those above the 75th percentile (≥125 months, approximately 10 years) to minimise potential bias from participants with longer intervals. The analysis was conducted using the most recent UK Biobank data release available at the time (August 2025), which automatically excludes participants who have withdrawn consent, according to UK Biobank governance. Compared with the original dataset (n = 14,840), this updated version excluded 19 participants, resulting in 14,821 eligible participants; after applying the temporal exclusion criterion, 11,117 participants remained.

We then repeated the full analytical workflow following the same pipeline used for the entire cohort, including data preparation, dimensionality reduction (FAMD), and clustering. All *post hoc* analyses were repeated accordingly, including outcome models, CMR associations, and mediation analyses, using the same covariate adjustment strategy applied in the main analysis.

*Statistical analysis*

Clusters were compared on clinical characteristics using chi-squared tests for categorical variables and ANOVA (or Kruskal-Wallis when applicable) for continuous variables. Pairwise comparisons were performed with independent t-tests (for normally distributed data) or Wilcoxon rank-sum tests (for skewed data) for numeric variables, along with chi-squared tests for categorical variables. A two-sided p < 0.05 with Bonferroni correction for multiple testing, where appropriate, was deemed statistically significant for all analyses. All analyses were conducted using Python 3.8.10 (Python Software Foundation, Delaware USA) and Scikit-learn version 0.23.2.

**Supplemental Results - Sensitivity analyses**

*Sensitivity analysis 1 (cardiomyopathy cases excluded)*

A total of 1,513 participants had HTN and cardiomyopathies, distributed as follows: cluster 1, 4.9%; cluster 2, 13.9%; and cluster 3, 6.54%. After excluding these individuals (n=13,327), associations between clusters and clinical outcomes remained unchanged (Table S10).

Cluster associations with CMR metrics (n = 10,873) and their contributions to outcomes were consistent with the main analyses (Figure S3), and similar mediation patterns were observed (Figure S4), particularly for MACE. However, for all-cause mortality, only LA function, biventricular stroke volumes, and cardiac mechanics remained significant mediators in both clusters, while LV geometric remodelling was no longer significant in cluster 2 (Figure S5). This suggests that including individuals with cardiomyopathies may have slightly overestimated the mediating role of LV remodelling in mortality, without meaningfully affecting other mediation results.

*Sensitivity analysis 2 (temporal heterogeneity)*

After excluding participants with long intervals between baseline and CMR assessments (≥125 months, approximately 10 years), a total of 11,117 participants remained for re-analysis. The same analytical pipeline used for the whole cohort was applied, including data preprocessing, FAMD for mixed data, and K-Means clustering (approach 2).

The FAMD step retained 40 principal components explaining over 80% of the total variance, and three clusters were again identified as the optimal solution based on a combination of quantitative metrics and clinical interpretability (elbow at $k=3$, inertia ≈100,000; silhouette values 0.097 for $k=2$and 0.088 for $k=3$; Figure S6), consistent with the original analysis.

Cluster composition and distribution closely mirrored the original findings: Cluster 1 (n = 1613) resembled the original Cluster 3, displaying intermediate cardiometabolic and imaging profiles; Cluster 2 (n = 6213) corresponded to the original Cluster 2, representing the highest-risk group with the most adverse cardiometabolic and imaging features; and Cluster 3 (n = 3291) resembled the original Cluster 1, showing the most favourable cardiovascular profile (Figure S7). Prevalence of comorbidities and clinical characteristics across clusters remained highly comparable to the main analysis, supporting reproducibility of the phenotypic structure (Table S11).

Repeating the *post hoc* analyses yielded consistent findings. Cluster-outcome associations (Table S12) confirmed that Cluster 2 (highest-risk profile) remained associated with the greatest risk for all adverse events, while Cluster 1 (intermediate group) showed a selective increase in incident AF, with attenuated risk for death. Cluster 3, similar to the original Cluster 1, remained the lowest-risk group. Associations between clusters and CMR metrics also paralleled the main results, with Cluster 2 showing the greatest adverse remodelling and impaired function, Cluster 3 the most favourable imaging profile, and Cluster 1 intermediate characteristics (Figure S8). Mediation analyses for major outcomes (MACE and AF; Figure S9, S10) demonstrated consistent indirect effects through CMR metrics, confirming similar patterns to the primary analysis. Overall, these findings indicate that the identified phenotypes and their clinical and imaging correlates were robust to temporal heterogeneity and dataset updates.

**Supplemental References**

1. Petersen SE, Matthews PM, Francis JM, Robson MD, Zemrak F, Boubertakh R, Young AA, Hudson S, Weale P, Garratt S, et al. UK Biobank’s cardiovascular magnetic resonance protocol. *Journal of Cardiovascular Magnetic Resonance*. 2016.

2. Petersen SE, Aung N, Sanghvi MM, Zemrak F, Fung K, Paiva JM, Francis JM, Khanji MY, Lukaschuk E, Lee AM, et al. Reference ranges for cardiac structure and function using cardiovascular magnetic resonance (CMR) in Caucasians from the UK Biobank population cohort. *Journal of Cardiovascular Magnetic Resonance*. 2017;19.

3. Bai W, Sinclair M, Tarroni G, Oktay O, Rajchl M, Vaillant G, Lee AM, Aung N, Lukaschuk E, Sanghvi MM, et al. Automated cardiovascular magnetic resonance image analysis with fully convolutional networks 08 Information and Computing Sciences 0801 Artificial Intelligence and Image Processing. *Journal of Cardiovascular Magnetic Resonance*. 2018;20.

4. Mewton N, Opdahl A, Choi EY, Almeida ALC, Kawel N, Wu CO, Burke GL, Liu S, Liu K, Bluemke DA, et al. Left ventricular global function index by magnetic resonance imaging - A novel marker for assessment of cardiac performance for the prediction of cardiovascular events: The multi-ethnic study of atherosclerosis. *Hypertension*. 2013;61:770–778.

5. Xia Y, Chen X, Ravikumar N, Kelly C, Attar R, Aung N, Neubauer S, Petersen SE, Frangi AF. Automatic 3D+t four-chamber CMR quantification of the UK biobank: integrating imaging and non-imaging data priors at scale. *Med Image Anal*. 2022;80.

6. Habibi M, Samiei S, Ambale Venkatesh B, Opdahl A, Helle-Valle TM, Zareian M, Almeida ALC, Choi E-Y, Wu C, Alonso A, et al. Cardiac Magnetic Resonance-Measured Left Atrial Volume and Function and Incident  Atrial Fibrillation: Results From MESA (Multi-Ethnic Study of Atherosclerosis). *Circ Cardiovasc Imaging*. 2016;9.

7. Badano LP, Miglioranza MH, MihǍilǍ S, Peluso D, Xhaxho J, Marra MP, Cucchini U, Soriani N, Iliceto S, Muraru D. Left atrial volumes and function by three-dimensional echocardiography: Reference values, accuracy, reproducibility, and comparison with two-dimensional echocardiographic measurements. *Circ Cardiovasc Imaging*. 2016;9.

8. Hann E, Popescu IA, Zhang Q, Gonzales RA, Barutçu A, Neubauer S, Ferreira VM, Piechnik SK. Deep neural network ensemble for on-the-fly quality control-driven segmentation of cardiac MRI T1 mapping. *Med Image Anal*. 2021;71.

9. Chadalavada S, Rauseo E, Salih A, Naderi H, Khanji M, Vargas JD, Lee AM, Amir-Kalili A, Lockhart L, Graham B, et al. Quality control of cardiac magnetic resonance imaging segmentation, feature tracking, aortic flow, and native T1 analysis using automated batch processing in the UK Biobank study. *European heart journal. Imaging methods and practice*. 2024; 2.

10. Chadalavada S, Fung K, Rauseo E, Lee AM, Khanji MY, Amir-Khalili A, Paiva J, Naderi H, Banik S, Chirvasa M, et al. Myocardial Strain Measured by Cardiac Magnetic Resonance Predicts Cardiovascular Morbidity and Death. *J Am Coll Cardiol*. 2024;84:648–659.

11. Biasiolli L, Hann E, Lukaschuk E, Carapella V, Paiva JM, Aung N, Rayner JJ, Werys K, Fung K, Puchta H, et al. Automated localization and quality control of the aorta in cine CMR can significantly accelerate processing of the UK Biobank population data. *PLoS One*. 2019;14.

12. Mansournia MA, Nazemipour M, Etminan M. A practical guide to handling competing events in etiologic time-to-event studies. Glob Epidemiol. 2022;4.

13. Austin PC, Lee DS, Fine JP. Introduction to the Analysis of Survival Data in the Presence of Competing Risks. *Circulation*. 2016;133.

14. Schuster NA, Hoogendijk EO, Kok AAL, Twisk JWR, Heymans MW. Ignoring competing events in the analysis of survival data may lead to biased results: a nonmathematical illustration of competing risk analysis. *J Clin Epidemiol*. 2020;122.

15. Hayes Andrew F. Introduction to Mediation, Moderation, and Conditional Process Analysis - Model Numbers. 2013.

16. VanderWeele TJ. Mediation Analysis: A Practitioner’s Guide. Annu Rev Public Health. 2016;37.

**Table S1. Inclusion and exclusion criteria for defining the hypertensive study cohort.**

| **Source** | | **UK Biobank field** | | **Description** |
| --- | --- | --- | --- | --- |
| ***Essential hypertension*** |  | |  | |
| Self-reported illness | | 20002 | | Hypertension |
| ICD10 Summary diagnoses | | 41270, 41280 | | I10 Essential (primary) hypertension |
| First occurrences | | 131286 | | Date I10 first reported (essential (primary) hypertension) |
| Diagnosed by doctor | | 6150  2966 | | High blood pressure  Age high blood pressure diagnosed |
| Medications | | (6153, 6177) :2 | | Blood pressure medication |
| ***Heart failure*** | |  | |  |
| Self-reported condition | | 20002 | | heart failure/pulmonary oedema |
| ICD10 Summary diagnoses | | 41270, 41280 | | I50.0 Heart failure  I50.1 Left ventricular failure  I50.9 Heart failure, unspecified |
|  | |  | | I11.0 Hypertensive heart disease with (congestive) heart failure  I13.0 Hypertensive heart and renal disease with (congestive) heart failure  I13.2 Hypertensive heart and renal disease with both (congestive) heart failure and renal failure  K76.1 Chronic passive congestion of liver  J81 Pulmonary oedema |
| First occurrences | | 131354 | | Date first diagnosis: Heart failure |
|  | | 131670 | | Date first diagnosis: Congestion of liver |
|  | | 131524 | | Date first diagnosis: Pulmonary oedema |

This table outlines the criteria for defining the hypertensive study cohort and the exclusion criteria for heart failure. All conditions must be met at the time of the imaging visit to ensure consistency in participant selection.

**Table S2. Phenotypic features scrutinised for clustering, with corresponding UK Biobank data fields.**

| **Phenotypic domains** | **Phenotypic features** | **UK Biobank field** |
| --- | --- | --- |
| ***Socio-demographics*** | | |
|  | Age | 21003 |
|  | Ethnicity*^*^* | 21000 |
|  | Sex^*^ | 31 |
|  | Education level^*^ | 6138 |
|  | Townsend deprivation index | 189 |
| ***Physical measurements*** | |  |
|  | Body Mass Index (BMI) | 21001 |
|  | Body Surface Area (BSA) | 50,21002 |
|  | Height | 50 |
|  | Weight | 21002 |
|  | Waist circumference (WC) | 48 |
|  | Hip circumference (HC) | 49 |
|  | Waist hip ratio (WHR) | 48, 49 |
|  | Waist height ratio (WHtR) | 48, 50 |
|  | Fat mass (FM) | 23100 |
|  | Fat free mass (FFM) | 23101 |
|  | Fat mass index (FMI) | 23100, 50 |
|  | Fat free mass index (FFMI) | 23101, 50 |
|  | Basal metabolism rate (BMR) | 23105 |
|  | Body fat percentage (BF) | 23099 |
|  | Systolic Blood Pressure (SBP) | 4080 |
|  | Diastolic Blood Pressure (DBP) | 4079 |
|  | Pulse wave arterial stiffness index (ASI) | 21021 |
|  | Pulse rate (when reading automated blood pressure) | 102 |
| ***Lifestyle factors*** | |  |
|  | Smoking^*^ | 20116 |
|  | Alcohol intake^*^ | 1558 |
|  | Physical activity – total METs | 864, 874, 884, 894, 904, 914 |
|  | Time watching TV, hours/day | 1070 |
|  | Time using computer, hours/day | 1080 |
|  | Sleep duration, hours/day | 1160 |
|  | Fruit intake, serving/day^*^ | 1309, 1319 |
|  | Vegetable intake, serving/day^*^ | 1299, 1289 |
|  | Oily fish intake, portion/week^*^ | 1329 |
|  | Non oily fish intake, portion/week^*^ | 1339 |
|  | Processed meat intake, portion/week^*^ | 1349 |
|  | Red meat intake, portion/week^*^ | 1369,1379, 1389 |
|  | Coffee intake, cups/day^*^ | 1498 |
|  | Tea intake, cups/day^*^ | 1488 |
| ***Laboratory*** |  |  |
|  | Calcium, mmol/L | 30680 |
|  | Creatinine, umol/L | 30700 |
|  | Urea, mmol/L | 30670 |
|  | Urate, umol/L | 30880 |
|  | eGFR, mL/min/1.73msq ^a^ | 21003, 31, 21000, 30700 |
|  | Aspartate aminotransferase, U/L (AST) | 30650 |
|  | Alanine aminotransferase, U/L (ALT) | 30620 |
|  | Alkaline phosphatase, U/L (ALP) | 30610 |
|  | Gamma glutamyltransferase, U/L (GGT) | 30730 |
|  | Albumin, g/L | 30600 |
|  | Total bilirubin, umol/L | 30840 |
|  | Red blood cell count, 10^12 cells/L (RBC) | 30010 |
|  | White blood cell count, 10^9 cells/L (WBC) | 30000 |
|  | Platelet count, 10^9 cells/L | 30080 |
|  | Mean corpuscular volume, femtolitres (MCV) | 30040 |
|  | Mean corpuscular haemoglobin, picograms (MCH) | 30050 |
|  | Mean corpuscular haemoglobin concentration, gr/dL (MCHC) | 30060 |
|  | Haematocrit percentage, % (HT) | 30030 |
|  | Haemoglobin concentration, gr/dL | 30020 |
|  | Red blood cell distribution width, % (RDW) | 30070 |
|  | Cholesterol, mmol/L | 30690 |
|  | HDL cholesterol, mmol/L | 30760 |
|  | LDL cholesterol, mmol/L | 30780 |
|  | Triglycerides, mmol/L | 30870 |
|  | Microalbumin in urine, mg/L | 30500 |
|  | Sodium in urine, mmol/L | 30530 |
|  | Potassium in urine, mmol/L | 30520 |
|  | Creatinine in urine, micromole/L | 30510 |
|  | C-reactive protein mg/L (CRP) | 30710 |
|  | Vitamin D, nmol/L | 30890 |
|  | Glycated haemoglobin (HbA1c), mmol/mol | 30750 |
| ***Electrocardiography*** | | |
|  | P duration, ms | 12338 |
|  | QRS duration, ms | 12340 |
|  | PQ interval, ms | 22330 |
|  | QT interval, ms | 22331 |
|  | QTc interval, ms | 22332 |
|  | RR interval, ms | 22333 |
|  | PP interval, ms | 22334 |
|  | P axis, degrees | 22335 |
|  | R axis, degrees | 22336 |
|  | T axis, degrees | 22337 |
|  | Ventricular rate, bpm | 12336 |

Asterisks (*) indicate categorical variables. ^a^ Glomerular Filtration Rate (GRF) was estimated using the EPI creatinine equation. Laboratory data were collected at baseline assessment, while the other variables were recorded at the first imaging visit.

**Table S3. UK Biobank data sources used to identify clinical outcomes.**

| **Source** | **UK Biobank field** | **Description** |
| --- | --- | --- |
| ***Heart Failure (all causes)*** |  |  |
| Self-reported condition | 20002 | Cardiomyopathy |
|  |  | heart failure/pulmonary oedema |
| ICD10 Summary diagnoses | 41270, 41280 | I50 Heart failure  I42 Cardiomyopathy  I43 Cardiomyopathy in diseases classified elsewhere |
|  |  | I11.0 Hypertensive heart disease with (congestive) heart failure  I13.0 Hypertensive heart and renal disease with (congestive) heart failure  I13.2 Hypertensive heart and renal disease with both (congestive) heart failure and renal failure  I25.5 Ischaemic cardiomyopathy  K76.1 Chronic passive congestion of liver  J81 Pulmonary oedema |
| First occurrences | 131354 | Date first diagnosis: Heart failure |
|  | 131338 | Date first diagnosis: Cardiomyopathy |
|  | 131340 | Date first diagnosis: Cardiomyopathy in diseases classified elsewhere |
|  | 131288 | Date first diagnosis: Hypertensive heart disease |
|  | 131292 | Date first diagnosis: Hypertensive heart and renal disease |
|  | 131306 | Date first diagnosis: Ischaemic cardiomyopathy |
|  | 131670 | Date first diagnosis: Congestion of liver |
|  | 131524 | Date first diagnosis: Pulmonary oedema |
| ***Myocardial infarction*** |  |  |
| Self-reported illness | 20002 | heart attack/myocardial infarction |
| ICD10 Summary diagnoses | 41270, 41280 | I21 Acute myocardial infarction |
|  |  | I22 Subsequent myocardial infarction |
|  |  | I23 Certain current complications following acute myocardial infarction |
|  |  | I24.1 Dressler's syndrome |
|  |  | I25.2 Old myocardial infarction |
| First occurrences | 131298 | Date first diagnosis: Acute myocardial infarction |
|  | 131300 | Date first diagnosis: Subsequent myocardial infarction |
|  | 131302 | Date first diagnosis: Certain current complications following acute myocardial infarction |
|  | 131304 | Date first diagnosis: Other acute ischaemic heart diseases |
|  | 131306 | Date first diagnosis: Chronic ischaemic heart disease |
| Diagnosed by doctor | 3894 | Age heart attack diagnosed |
| Algorithmically defined | 42000 | Date of myocardial infarction |
| ***Stroke*** |  |  |
| Self-reported illness | 20002 | stroke |
| ICD10 Summary diagnoses | 41270, 41280 | I60 Subarachnoid haemorrhage |
|  |  | I61 Intracerebral haemorrhage |
|  |  | I62 Other nontraumatic intracranial haemorrhage |
|  |  | I63 Cerebral infarction |
|  |  | I64 Stroke, not specified as haemorrhage or infarction |
| First occurrences | 131360 | Date first diagnosis: Subarachnoid haemorrhage |
|  | 131362 | Date first diagnosis: Intracerebral haemorrhage |
|  | 131364 | Date first diagnosis: Other nontraumatic intracranial haemorrhage |
|  | 131366 | Date first diagnosis: Cerebral infarction |
|  | 131368 | Date first diagnosis: Stroke, not specified as haemorrhage or infarction |
| Diagnosed by doctor | 4056 | Age stroke diagnosed |
| Algorithmically defined | 42006 | Date of stroke |
| ***Peripheral vascular diseases*** |  |  |
| Self-reported illness | 20002 | peripheral vascular disease  aortic aneurysm |
| ICD10 Summary diagnoses | 41270, 41280 | I70 Atherosclerosis |
|  |  | I71 Aortic aneurysm and dissection |
|  |  | I72 Other aneurysm |
|  |  | I73.9 Peripheral vascular disease, unspecified |
| First occurrences | 131382 | Date first diagnosis: Aortic aneurysm and dissection |
|  | 131384 | Date first diagnosis: Other aneurysm |
|  | 131386 | Date first diagnosis: Other peripheral vascular diseases |
| ***Atrial fibrillation/flutter*** |  |  |
|  |  | atrial fibrillation |
| Self-reported illness | 20002 | atrial flutter |
| ICD10 Summary diagnoses | 41270, 41280 | I48.0 Paroxysmal atrial fibrillation |
|  |  | I48.1 Persistent atrial fibrillation |
|  |  | I48.2 Chronic atrial fibrillation |
|  |  | I48.3 Typical atrial flutter |
|  |  | I48.4 Atypical atrial flutter |
|  |  | I48.9 Atrial fibrillation and atrial flutter, unspecified |
|  |  |  |
| First occurrences | 131350 | Date first diagnosis: Atrial fibrillation and flutter |
| ***Death (any cause)*** |  |  |
| Death register | 40000 | Date of death |
| ***Cardiovascular death*** |  |  |
| Underlying (primary) cause of death: ICD-10 | 40001 | Diseases of the circulatory system: I05-I89 |

**Table S4. Baseline clinical characteristics of the study cohort.**

| **Baseline characteristics** | **All cohort**  **(n = 14,840)** |
| --- | --- |
| ***Demographics*** | |
| Age, years | 66.56±7.24 |
| Female, n (%) | 6217(41.89) |
| Ethnicity, n (%) |  |
| *White* | 14368(96.82) |
| *Others* | 471(3.17) |
| ***Comorbidities, n (%)*** | |
| Smoking history | 2586(17.42) |
| Previous myocardial infarction | 1608(10.83) |
| Stroke | 585(3.94) |
| Chronic obstructive pulmonary disease | 391(2.63) |
| Asthma | 2216(14.93) |
| Atrial fibrillation | 736(4.96) |
| Peripheral artery disease | 319(2.15) |
| Diabetes | 1790(12.06) |
| Hypercholesterolemia | 7898(53.22) |
| CKD | 657(4.43) |
| ***Physical measurements*** | |
| Body mass index, Kg/m^2^ | 27.48(25.03-30.46) |
| Waist hip ratio | 0.89±0.08 |
| Systolic blood pressure, mmHg | 147.08 ±18.3 |
| Diastolic blood pressure, mmHg | 81.51±10.28 |
| ***Laboratories*** | |
| eGFR, mL/min/1.73m² | 89.98 ±12.81 |
| LDL cholesterol, mmol/L | 3.52 ±0.87 |
| Triglycerides, mmol/L | 1.60(1.14-2.27) |

Values are mean (±standard deviation) when continuous or number (percentage) when categorical. Data are presented as median (interquartile range) where absolute skew is ≥ 0.9*.*

**Table S5. Health-related lifestyle behaviours according to the identified clusters.**

|  | **Cluster 1**  **(n=4300)** | **Cluster 2**  **(n=8339)** | **Cluster 3 (n=2201)** | **P Value** | **Cluster 1 vs Cluster 2** | **Cluster 1 vs Cluster 3** | **Cluster 2 vs Cluster 3** |
| --- | --- | --- | --- | --- | --- | --- | --- |
| ***Lifestyle habits*** | | | | |  |  |  |
| Smoking history | 1567(36.44) | 4118(49.38) | 919(41.75) | <0.001 | <0.001 | <0.001 | <0.001 |
| Alcohol intake, frequency |  |  |  | <0.001 | <0.001 | <0.001 | <0.001 |
| *Never* | 233(5.42) | 290(3.48) | 186(8.45) |  |  |  |  |
| *Special occasion only* | 403(9.37) | 393(4.71) | 446(20.26) |  |  |  |  |
| *1-3 times a month* | 503(11.69) | 623(7.47) | 352(15.99) |  |  |  |  |
| *1-2 times a week* | 1073(24.95) | 1946(23.45) | 545(24.76) |  |  |  |  |
| *3-4 times a week* | 1124(26.14) | 2501(29.99) | 390(17.72) |  |  |  |  |
| *Daily or almost daily* | 964(22.42) | 2576(30.81) | 282(12.81) |  |  |  |  |
| Time watching TV, hours/day | 2.47±1.42 | 2.60±1.47 | 3.03±1.63 | <0.001 | <0.001 | <0.001 | <0.001 |
| Time using computer, hours/day | 0.99±1.11 | 1.53±1.49 | 1.26±1.46 | <0.001 | <0.001 | <0.001 | <0.001 |
| Sleep duration, hours/day | 7.18±0.99 | 7.15±1 | 7.16±1.14 | 0.24 |  |  |  |
| Physical activity -Total METs | 1910(1124-3600) | 1892(996-3433) | 1644(714-2678) | <0.001 | <0.001 | <0.001 | <0.001 |
| ***Dietary habits*** | | | | |  |  |  |
| Fruits intake, servings per day |  |  |  | <0.001 | <0.001 | <0.001 | <0.001 |
| *< 3 servings a day* | 2073(48.21) | 5183(62.15) | 1261(57.29) |  |  |  |  |
| *3 - 4 servings a day* | 1018(23.67) | 1455(17.44) | 471(21.40) |  |  |  |  |
| *4-6 servings a day* | 885(20.58) | 1257(15.07) | 370(16.81) |  |  |  |  |
| *≥6 serving a day* | 324(7.53) | 444(5.32) | 99(4.49) |  |  |  |  |
| Vegetables intake, servings per day |  |  |  | <0.001 | <0.001 | 0.11 | <0.001 |
| *< 3 servings a day* | 2813(65.42) | 6021(72.20) | 1495(67.92) |  |  |  |  |
| *3 - 4 servings a day* | 880(20.46) | 1336(16.02) | 448(20.35) |  |  |  |  |
| *4-6 servings a day* | 484(11.25) | 734(8.80) | 199(9.04) |  |  |  |  |
| *≥6 serving a day* | 123(2.86) | 248(2.97) | 59(2.68) |  |  |  |  |
| Oily fish intake, frequency |  |  |  | <0.001 | 1.46 | <0.001 | <0.001 |
| *0 -1 time/week* | 353(8.20) | 642(7.70) | 253(11.49) |  |  |  |  |
| *1-2 times/week* | 3118(72.51) | 6120(73.39) | 1585(72.01) |  |  |  |  |
| *≥ 2 times/week* | 829(19.28) | 1577(18.91) | 363(16.49) |  |  |  |  |
| Non oily fish intake, frequency |  |  |  | <0.001 | <0.001 | 0.81 | 0.007 |
| *0 -1 time/week* | 194(4.51) | 253(3.03) | 96(4.36) |  |  |  |  |
| *1-2 times/week* | 3440(80) | 6759(81.05) | 1730(78.60) |  |  |  |  |
| *≥ 2 times/week* | 666(15.49) | 1327(15.91) | 375(17.04) |  |  |  |  |
| Processed food intake, frequency |  |  |  | <0.001 | <0.001 | <0.001 | <0.001 |
| *0 -1 time/week* | 583(13.56) | 345(4.14) | 151(6.86) |  |  |  |  |
| *1-2 times/week* | 2987(69.46) | 4469(53.59) | 1449(65.83) |  |  |  |  |
| *≥ 2 times/week* | 730(16.98) | 3525(42.27) | 601(27.30) |  |  |  |  |
| Red meat intake, frequency |  |  |  | <0.001 | <0.001 | <0.001 | <0.001 |
| *0 -1 time/week* | 614(14.35) | 536(6.43) | 201(9.13) |  |  |  |  |
| *1-2 times/week* | 3119(72.53) | 6424(77.03) | 1610(73.14) |  |  |  |  |
| *2-3 times/week* | 88(2.04) | 144(1.73) | 80(3.63) |  |  |  |  |
| *≥ 4 times/week* | 476(11.07) | 1235(14.81) | 310(14.08) |  |  |  |  |
| Coffee intake, cups per day |  |  |  | <0.001 | <0.001 | <0.001 | 0.003 |
| *0-1 cup/day* | 2205(51.28) | 3681(44.14) | 1064(48.34) |  |  |  |  |
| *1-2 cups/day* | 880(20.46) | 1623(19.46) | 375(17.03) |  |  |  |  |
| *≥ 3 cups/day* | 1215(28.25) | 3035(36.39) | 772(34.62) |  |  |  |  |
| Tea intake, cups per day |  |  |  | <0.001 | <0.001 | 0.53 | <0.001 |
| *0-1 cup/day* | 1022(23.77) | 2178(26.11) | 686(31.17) |  |  |  |  |
| *1-3 cups/day* | 631(14.67) | 1236(14.82) | 303(13.77) |  |  |  |  |
| *≥ 3 cups/day* | 2647(61.56) | 4925(59.06) | 1212(55.07) |  |  |  |  |

Categorical values are presented as count (percentage); continuous values are presented as mean (±standard deviation) and as median (interquartile range) where absolute skew ≥ 0.9. The P Value indicates comparisons of variables across clusters and bold values indicate statistical significance (p <0.05).

**Table S6. Laboratory and electrocardiographic characteristics stratified by clusters.**

|  | **Cluster 1**  **(n=4300)** | **Cluster 2**  **(n=8339)** | **Cluster 3**  **(n=2201)** | **P Value** | **Cluster 1 vs Cluster 2** | **Cluster 1 vs Cluster 3** | **Cluster 2 vs Cluster 3** |
| --- | --- | --- | --- | --- | --- | --- | --- |
| ***Laboratories*** | | | | |  |  |  |
| Calcium, mmol/L | 2.39±0.09 | 2.37±0.08 | 2.38±0.09 | <0.001 | <0.001 | <0.001 | <0.001 |
| Creatinine, umol/L | 64.1(57.8-72.3) | 79.8(73.5-88.3) | 65.1(58.7-73.5) | <0.001 | <0.001 | <0.001 | <0.001 |
| Urea, mmol/L | 5.26(4.51-5.9) | 5.4(4.8-6.24) | 5.37(4.56-6.06) | <0.001 | <0.001 | <0.001 | <0.001 |
| Urate, umol/L | 269±58.4 | 360±67.83 | 319±66.13 | <0.001 | <0.001 | <0.001 | <0.001 |
| eGFR, mL/min/1.73m² | 90.7±11.96 | 89.39±12.30 | 90.71±13.31 | <0.001 | <0.001 | 1 | <0.001 |
| Aspartate aminotransferase, U/L | 23.6(20.5-26.6) | 26(23.3-30.7) | 24.3(20.7-27.5) | <0.001 | <0.001 | <0.001 | <0.001 |
| Alanine aminotransferase, U/L | 17.8(14.1-22.1) | 24.4(19.9-32.8) | 22.1(17.2-28.4) | <0.001 | <0.001 | <0.001 | <0.001 |
| Alkaline phosphatase, U/L | 79.9(67.9-94.2) | 79.1(67.3-89.9) | 86.5(76.7-102.9) | <0.001 | <0.001 | <0.001 | <0.001 |
| Gamma glutamyl transferase, U/L | 21.8(16.5-29.3) | 33.4(25.8-49.5) | 29.3(21.2-41.2) | <0.001 | <0.001 | <0.001 | <0.001 |
| Albumin, g/L | 45.4±2.34 | 45.7±2.30 | 44.6±2.29 | <0.001 | <0.001 | <0.001 | <0.001 |
| Total bilirubin, umol/L | 7.5(5.7-10.1) | 10.1(8.2-11.9) | 9.3(7.5-11.3) | <0.001 | <0.001 | <0.001 | 0.001 |
| White blood cell count, 10^9 cells/L | 6.4(5.5-7.3) | 6.6(5.7-7.6) | 7.1(6.2-8.4) | <0.001 | <0.001 | 0.001 | <0.001 |
| Platelet count, 10^9 cells/L | 260±55.5 | 236±51.6 | 258±58.1 | <0.001 | <0.001 | <0.001 | <0.001 |
| Mean corpuscular haemoglobin, pg | 31.5±1.71 | 31.69±1.58 | 30.76±1.88 | <0.001 | <0.001 | <0.001 | <0.001 |
| Mean corpuscular haemoglobin concentration, g/dl | 34.5(33.9-35) | 34.6(34.1-35.3) | 34.4(33.8-34.9) | <0.001 | <0.001 | <0.001 | <0.001 |
| Haemoglobin, g/dl | 13.65±0.92 | 15.07±0.94 | 13.71±1.02 | <0.001 | <0.001 | 0.038 | <0.001 |
| Red blood cell distribution width, % | 13.3(12.9-13.7) | 13.3(12.9-13.7) | 13.4(13-14.01) | <0.001 | 0.02 | <0.001 | <0.001 |
| Total cholesterol, mmol/L | 5.89±1.05 | 5.45±1.12 | 5.74±1.10 | <0.001 | <0.001 | <0.001 | <0.001 |
| HDL cholesterol, mmol/L | 1.62±0.36 | 1.28±0.28 | 1.37±0.28 | <0.001 | <0.001 | <0.001 | <0.001 |
| LDL cholesterol, mmol/L | 3.61±0.81 | 3.45±0.85 | 3.62±0.84 | <0.001 | <0.001 | 1 | <0.001 |
| Triglycerides, mmol/L | 1.33(0.97-1.69) | 1.68(1.27-2.43) | 1.71(1.37-2.31) | <0.001 | <0.001 | <0.001 | 0.40 |
| Sodium in urine, mmol/L | 59.6±35.22 | 85.75±43.04 | 81.19±43.96 | <0.001 | <0.001 | <0.001 | <0.001 |
| Potassium in urine, mmol/L | 55.08±31.14 | 69.34±33.43 | 65.28±33.16 | <0.001 | <0.001 | <0.001 | <0.001 |
| Creatinine in urine, mmol/L | 5002(3152-7985) | 9685(6278-13956) | 7863(4719-11613) | <0.001 | <0.001 | <0.001 | <0.001 |
| C-reactive protein, mg/L | 1.09(0.55-1.85) | 1.32(0.71-2.18) | 2.79(1.38-5.15) | <0.001 | <0.001 | <0.001 | <0.001 |
| Vitamin D, nmol/L | 52.22±19.64 | 49.62±20.05 | 42.70±16.8 | <0.001 | <0.001 | <0.001 | <0.001 |
| Glycated haemoglobin, mmol/mol | 35.2(32.8-37) | 35.4(33.1-37.7) | 36.3(34.3-39.6) | <0.001 | <0.001 | <0.001 | <0.001 |
| ***Resting 12 leads ECG*** | | | | |  |  |  |
| P duration, ms | 98.11±14.62 | 100.09±16.04 | 97.09±15.10 | <0.001 | <0.001 | 0.026 | <0.001 |
| QRS duration, ms | 86(78-88) | 90(86-98) | 86(80-90) | <0.001 | <0.001 | 0.021 | <0.001 |
| QT interval, ms | 420.52±30.62 | 417.60±31.03 | 414.57±32.59 | <0.001 | <0.001 | <0.001 | <0.001 |
| QTc interval, ms | 424(416-441) | 424(406-432) | 428(420-445) | <0.001 | <0.001 | <0.001 | <0.001 |
| PP interval, ms | 962.4±166.41 | 983.02±186.72 | 921.71±158.91 | <0.001 | <0.001 | <0.001 | <0.001 |
| RR interval, ms | 968(878-1046) | 968(894-1086) | 940(822-1000) | <0.001 | <0.001 | <0.001 | <0.001 |
| PQ interval, ms | 166(150-174) | 166(158-184) | 166(148-174) | <0.001 | <0.001 | 0.52 | <0.001 |
| P axis, degrees | 50.34±21.21 | 48.39±21.18 | 46.31±21.09 | <0.001 | <0.001 | <0.001 | <0.001 |
| R axis, degrees | 26.73±33.52 | 18±35.30 | 18.48±29.37 | <0.001 | <0.001 | <0.001 | 1 |
| T axis, degrees | 39(30-57) | 38(20-50) | 38(23-50) | <0.001 | <0.001 | <0.001 | 0.32 |
| Ventricular rate, bpm | 62(57-68) | 62(55-67) | 64(60-73) | <0.001 | <0.001 | <0.001 | <0.001 |

Categorical values are presented as count (percentage); continuous values are presented as mean (±standard deviation) and as median (interquartile range) where absolute skew ≥ 0.9. The P Value indicates comparisons of variables across clusters and bold values indicate statistical significance (p <0.05).

*.*

**Table S7. Association of clusters with adverse outcomes on Cox proportional hazards analysis – sensitivity analysis (cases with less than 6 months follow-up have been excluded).**

|  | **Cluster 1**  **(n=4265)** | **Cluster 2**  **(n=8210)** | **Cluster 3**  **(n=2174)** |
| --- | --- | --- | --- |
| ***Model 1 (unadjusted), HR (95% CI)*** |  |  |  |
| Incident HF, n=14,649 | 1 | 2.10 (1.66 - 2.66)** | 1.37 (0.99 - 1.89) |
| Incident AF, n=13,951 | 1 | 2.09 (1.56 - 2.81) ** | 1.68(1.14 -2.46) * |
| Vascular Atherosclerotic events, n=12,411 | 1 | 2.03 (1.60 - 2.57) ** | 1.34 (0.97 - 1.85) |
| Death for all causes, n=14,649 | 1 | 2.15 (1.51 - 3.07) ** | 1.68 (1.05 - 2.68) * |
| Non-CVD death unadjusted |  | 1.92 (1.30 - 2.83) ** | 1.63 (0.98 - 2.73) |
| Combined MACE, n=11,960 | 1 | 1.86 (1.54 - 2.24) ** | 1.36 (1.05 - 1.76) * |
| Non-CVD death |  | 1.92 (1.30 - 2.83) ** | 1.63 (0.98 - 2.73) |
| ***Model 2 (adjusted), HR (95% CI)*** |  |  |  |
| Incident HF, n=14,649 | 1 | 1.75 (1.37 - 2.22) ** | 1.21 (0.87 - 1.69) |
| Incident AF, n=13,951 | 1 | 1.89 (1.40 - 2.56) ** | 1.56 (1.05 -2.30) * |
| Vascular Atherosclerotic events, n=12,411 | 1 | 1.87 (1.47 - 2.37) ** | 1.23 (0.88 - 1.70) |
| Death for all causes | 1 | 1.82 (1.27 - 2.62) ** | 1.50 (0.94 - 2.41) |
| Combined MACE, n=11,960^a^ | 1 | 1.79 (1.47 - 2.16) ** | 1.28 (0.98 - 1.66) |
| Non-CVD death |  | 1.55 (1.04 - 2.31) * | 1.40 (0.83 - 2.36) |

– *Model 1 (unadjusted):* clusters only.
– *Model 2 (adjusted):* clusters + covariates.
For incident HF, no prior HF cases existed by inclusion criteria; adjusted models include a composite prior-CVD covariate (prior MI, stroke, PAD, or AF), weighted as 1 or 0, alongside diabetes and hypercholesterolemia.
For incident AF, prevalent AF cases were excluded; adjusted models include prior-CVD composite, diabetes, and hypercholesterolemia.
For vascular atherosclerotic events, prevalent MI, stroke, or PAD cases were excluded; adjusted models include prior-CVD composite, diabetes, and hypercholesterolemia.
For MACE, all prior CVDs were excluded; adjusted models include only diabetes and hypercholesterolemia due to convergence limitations when adjusting for composite prior-CVD.
For all-cause, non‑CVD death, and CVD death, no CVD exclusions were made; adjusted models include the prior-CVD composite, diabetes, and hypercholesterolemia.
Abbreviations: AF = atrial fibrillation; CI = confidence interval; HF = heart failure; HR = hazard ratio; MI = myocardial infarction; PAD = peripheral artery disease; CVD = cardiovascular disease; MACE = major adverse cardiovascular events. *p < 0.05; **p < 0.005.

**Table S8. Summary of Cox model performances.**

| **Outcome** | **Unadjusted pseudo-R²** | **Covariates pseudo-R²** | **Full Model pseudo-R²** | **Incremental pseudo-R² (clusters)** | **Unadjusted**  **Concordance** | **Covariates Concordance** | **Full Model Concordance** |
| --- | --- | --- | --- | --- | --- | --- | --- |
| **Incident HF, n=14,840** | 0.0073 | 0.0147 | 0.0187 | 0.0041 | 0.575 | 0.615 | 0.638 |
| **Incident AF, n=14,104** | 0.0058 | 0.0079 | 0.0119 | 0.0041 | 0.573 | 0.575 | 0.617 |
| **Vascular Atherosclerotic events, n=12,532** | 0.0076 | 0.0040 | 0.0102 | 0.0062 | 0.577 | 0.550 | 0.595 |
| **Death for all causes, n=14,840** | 0.0049 | 0.0096 | 0.0123 | 0.0026 | 0.568 | 0.597 | 0.621 |
| **Combined MACE, n=12,057** | 0.0062 | 0.0018 | 0.0072 | 0.0055 | 0.566 | 0.532 | 0.575 |

**Table S9. Associations of CMR metrics with clustering.**

|  | **Cluster 2** | | **Cluster 3** | | |
| --- | --- | --- | --- | --- | --- |
|  | ***Beta coefficient [95% CI]*** | ***p value*** | ***Beta coefficient***  ***[95% CI]*** | ***p value*** |  |
| ***CMR indices of cardiac structure and function*** | | | | | |
| LVEDV, indexed (ml/m^2^) | 8.67 [8.14, 9.21] | <0.001 | -2.03 [-2.78, -1.28] | <0.001 |  |
| LVESV, indexed (ml/m^2^) | 5.94 [5.60, 6.28] | <0.001 | -0.78 [-1.25, -0.31] | 0.001 |  |
| LVSV, indexed (ml/m^2^) | 2.72 [2.38, 3.06] | <0.001 | -1.25 [-1.71, -0.78] | <0.001 |  |
| LVM, indexed (g/m^2^) | 9.68 [9.37, 9.99] | <0.001 | 0.14 [-0.28, 0.57] | 0.511******* |  |
| LVEF (%) | -3.15 [-3.40, -2.90] | <0.001 | -0.02 [-0.38, 0.33] | 0.808******* |  |
| RVEDV, indexed (ml/m^2^) | 12.27 [11.7, 12.8] | <0.001 | -1.75 [-2.53, -0.98] | <0.001 |  |
| RVESV, indexed (ml/m^2^) | 8.42 [8.09, 8.75] | <0.001 | -0.52 [-0.98, -0.06] | 0.025***** |  |
| RVSV, indexed (ml/m^2^) | 3.84 [3.49, 4.19] | <0.001 | -1.23 [-1.72, -0.74] | <0.001 |  |
| RVEF (%) | -3.97 [-4.21, -3.72] | <0.001 | -0.31 [-0.65, -0.03] | 0.07***** |  |
| M/V ratio (g/ml) | 0.06 [0.05, 0.06] | <0.001 | 0.02[ 0.01, 0.02] | <0.001 |  |
| Global LV wall thickness | 1.60 [1.54, 1.65] | <0.001 | 0.43[ 0.36, 0.51] | <0.001 |  |
| LVGFI (%) | 0.08 [-0.20, 0.36] | 0.546***** | -0.11 [-0.51, 0.29] | 0.598***** |  |
| Myocardium native T1 (ms) | -19.55 [-21.02, -18] | <0.001 | -2.18 [-4.22, -0.14] | 0.036******* |  |
| ***CMR indices of atrial structure and function*** | | | | | |
| LAV max, indexed(mL/m^2^) | -3.46 [-3.83, -3.09] | <0.001 | -2.34 [-2.87, -1.83] | <0.001 |  |
| LAV min, indexed(mL/m^2^) | -1.10 [-1.30, -0.90] | <0.001 | -0.69 [-0.99, -0.41] | <0.001 |  |
| LAV pre-atrial contraction, indexed (mL/m^2^) | -1.75 [-2.04, -1.47] | <0.001 | -1.34 [-1.73, -0.94] | <0.001 |  |
| Total LAEF (%) | -1.25 [-1.59, -0.92] | <0.001 | -1.19 [-1.66, 0.782 | <0.001 |  |
| Active LAEF (%) | 0.40 [-0.02, 0.77] | 0.038******* | -0.80 [-1.33, -0.27] | 0.003 |  |
| Passive LAEF (%) | -3.05 [-3.43, -2.76] | <0.001 | -1.31 [-1.72, -0.91] | <0.001 |  |
| Left atrium expansion index (%) | -0.13 [-0.16, -0.10] | <0.001 | -0.12 [-0.16, -0.08] | <0.001 |  |
| ***CMR indices of arterial function*** | | | | | |
| AoD, AA (x10^-3^/mmHg) | 0.23 [0.09, 0.37] | <0.001 | 0.21 [0.01, 0.40] | 0.033******* |  |
| AoD, DA (x10^-3^/mmHg) | 0.08 [-0.13, 0.30] | 0.417******* | 0.27 [-0.03, 0.57] | 0.070***** |  |
| SVR, indexed (mmHg.min.L^−1^.m^2^) | 10.50 [3.03, 17.0] | 0.005 | 3.29 [-7.14, 13.71] | 0.537***** |  |
| TAC, indexed (mL/mmHg/m^2^) | 0.08 [0.01, 0.14] | 0.029******* | -0.01 [-0.11, 0.86] | 0.825***** |  |
| ***CMR indices of cardiac mechanics*** | | | | | |
| LV GLS (%) | -1.01 [-1.10, -0.92] | <0.001 | 0.11 [-0.01, 0.24] | 0.077******* |  |
| LV GCS (%) | -1.15 [-1.24, -1.05] | <0.001 | -0.23 [-0.36, -0.10] | <0.001 |  |
| LV GRS (%) | -3.07 [-3.31, -2.83] | <0.001 | -0.61 [-0.94, -0.27] | <0.001 |  |
| LV Torsion (degrees) | -0.08 [-0.10, -0.06] | <0.001 | 0.02 [-0.006, 0.05] | 0.117******* |  |
| RV GLS (%) | -1.41 [-1.54, -1.27] | <0.001 | 0.28 [0.08, 0.47] | 0.003 |  |
| RV GCS (%) | 0.62 [0.49, 0.74] | <0.001 | 0.73 [0.56, 0.91] | <0.001 |  |
| RV GRS (%) | 1.60 [1.30, 1.89] | <0.001 | 1.67 [1.26, 2.08] | <0.001 |  |
| RV Torsion (degrees) | -0.41 [-0.45, -0.37] | <0.001 | 0.04 [-0.01, 0.09] | 0.121 ***** |  |

The non-standardised beta coefficient indicates the change in the CMR metric when comparing clusters 2 and 3 with cluster 1, which acts as the reference group. Asterisk (*****) denotes that the result is not significant after adjusting for multiple tests using Bonferroni correction (p < 0.0167). The strains are reported as absolute values, with higher values indicating more deformation regardless of the sign. AA = ascending aorta, AoD = aortic distensibility, DA = descending aorta, GCS = global circumferential strain, GLS = global longitudinal strain, GRS = global radial strain, LAEF = left atrial emptying fraction, LAV = left atrial volume; LVEDV = left ventricular end-diastolic volume, LVEF = left ventricular ejection fraction, LVM = left ventricular mass, LVESV = left ventricular end-systolic volume, LVGFI = left ventricle global function index, LVSV= left ventricular stroke volume, M/V = LV mass-to-volume ratio, RVEDV = right ventricular end-diastolic volume, RVEF = right ventricular ejection fraction, RVESV = right ventricular end-systolic volume, RVSV = right ventricular stroke volume, SVR = systemic vascular resistance, TAC= total arterial compliance.

**Table S10. Association of clusters with adverse outcomes on Cox proportional hazards analysis – sensitivity analysis (cardiomyopathy cases are excluded).**

|  | **Cluster 1**  **(n=4088)** | **Cluster 2**  **(n=7182)** | **Cluster 3**  **(n=2057)** | **P Value** |
| --- | --- | --- | --- | --- |
| ***Outcome, n (%)*** |  |  |  |  |
| Incident HF | 97(2.37) | 353(4.91) | 61(2.96) | <0.001 |
| Incident AF | 56(1.37) | 199(2.77) | 49(2.38) | <0.001 |
| Vascular Atherosclerotic events | 126(3.08) | 432(6.01) | 81(3.94) | <0.001 |
| Death for all causes | 37(0.90) | 130(1.81) | 34(1.65) | <0.001 |
| Combined MACE | 214(5.23) | 696(9.69) | 144(7) | <0.001 |
| ***Model 1 (unadjusted), HR (95% CI)*** |  |  |  |  |
| Incident HF, n=13,327 | 1 | 2.10 (1.68 - 2.63)** | 1.24(0.90 - 1.71) | … |
| Incident AF, n=12,779 | 1 | 2.09 (1.55 - 2.81) ** | 1.74 (1.19 - 2.55) ** |  |
| Vascular Atherosclerotic events, n=12,502 | 1 | 2.04 (1.65 - 2.54)** | 1.26 (0.93 - 1.71) | … |
| Death for all causes, n=13,327 | 1 | 2.01 (1.40 - 2.90) ** | 1.81 (1.14 - 2.89) * | … |
| Combined MACE, n=12,032 | 1 | 1.83 (1.53 - 2.18) ** | 1.31 (1.03 - 1.67) * | … |
| ***Model 2 (adjusted), HR (95% CI)*** |  |  |  |  |
| Incident HF, n=13,327 | 1 | 1.81 (1.44 - 2.27)** | 1.11 (0.80 - 1.53) | … |
| Incident AF, n=12,779 | 1 | 2(1.48 - 2.71) ** | 1.64(1.11 -2.42) * |  |
| Vascular Atherosclerotic events, n=12,502 | 1 | 1.90 (1.53 - 2.37)** | 1.17(0.86 - 1.59) | … |
| Death for all causes, n=13,327 | 1 | 1.80(1.24 - 2.61)** | 1.67 (1.04 - 2.68)* | … |
| Combined MACE, n=12,032 | 1 | 1.76 (1.47 - 2.11) ** | 1.24 (0.97 - 1.58) | … |

– *Model 1 (unadjusted):* clusters only.
– *Model 2 (adjusted):* clusters + covariates.
For incident HF, no prior HF cases existed by inclusion criteria; adjusted models include a composite prior-CVD covariate (prior MI, stroke, PAD, or AF), weighted as 1 or 0, alongside diabetes and hypercholesterolemia.
For incident AF, prevalent AF cases were excluded; adjusted models include prior-CVD composite, diabetes, and hypercholesterolemia.
For vascular atherosclerotic events, prevalent MI, stroke, or PAD cases were excluded; adjusted models include prior-CVD composite, diabetes, and hypercholesterolemia.
For MACE, all prior CVDs were excluded; adjusted models include only diabetes and hypercholesterolemia due to convergence limitations when adjusting for composite prior-CVD.
For all-cause, non‑CVD death, and CVD death, no CVD exclusions were made; adjusted models include the prior-CVD composite, diabetes, and hypercholesterolemia.
Abbreviations: AF = atrial fibrillation; CI = confidence interval; HF = heart failure; HR = hazard ratio; MI = myocardial infarction; PAD = peripheral artery disease; CVD = cardiovascular disease; MACE = major adverse cardiovascular events. *p < 0.05; **p < 0.005.

**Table S11. Main clinical characteristics stratified by cluster - sensitivity sample after excluding participants with long intervals between baseline and CMR assessment (temporal heterogeneity).**

|  | **Cluster 1**  **(n=1613)** | **Cluster 2**  **(n=6213)** | **Cluster 3**  **(n=3291)** | **P Value** |
| --- | --- | --- | --- | --- |
| ***Socio-demographics*** | | | | |
| Age, years | 64.35 ± 7.2 | 66.15 ± 7.08 | 65.9 ± 6.95 | <0.001 |
| Female, n (%) | 1498 (93 %) | 6 (0.09%) | 3149 (96%) | <0.001 |
| ***Comorbidities, n (%)*** | | | | |
| Previous myocardial infarction | 110 (6.8%) | 911 (14.7%) | 175 (5.3%) | <0.001 |
| Stroke | 60 (3.7%) | 275 (4.4%) | 90 (2.7%) | <0.001 |
| Atrial fibrillation | 61 (3.8%) | 363 (5.8%) | 93 (2.8%) | <0.001 |
| Peripheral artery disease | 29 (1.8%) | 137 (2.2%) | 84 (2.6%) | 0.232 |
| Diabetes | 355 (22.0%) | 826 (13.3%) | 152 (4.6%) | <0.001 |
| Hypercholesterolemia | 804 (49.8%) | 3772 (60.7%) | 1277 (38.8%) | <0.001 |

Categorical values are presented as count (percentage); continuous values are presented as mean (±standard deviation). The P Value indicates comparisons of variables across clusters.`

**Table S12. Association of clusters with adverse outcomes on Cox proportional hazards analysis -sensitivity analysis (temporal heterogeneity).**

|  | **Cluster 1**  **(n=1613)** | **Cluster 2**  **(n=6213)** | **Cluster 3**  **(n=3291)** | **P Value** |  |
| --- | --- | --- | --- | --- | --- |
| ***Outcome, n (%)*** |  |  |  |  |  |
| Incident HF | 59(3.7) | 354(5.7) | 98(3.0) | <0.001 |  |
| Incident AF | 47(2.9) | 215(3.5) | 49(1.5) | <0.001 |  |
| Vascular Atherosclerotic events | 77(4.8) | 458(7.4) | 131(4.0) | <0.001 |  |
| Death for all causes | 28(1.7) | 144(2.3) | 37(1.1) | <0.001 |  |
| Combined MACE | 140(8.7) | 764(12.3) | 216(6.6) | <0.001 |  |
| ***Model 1 (unadjusted),***  ***HR (95% CI)*** |  |  |  |  |  |
| Incident HF (n=11,117) | 1.23 (0.89 - 1.70) | 1.95 (1.56 - 2.44)** | 1 | … |  |
| Incident AF (n=10,600) | 1.99 (1.33 -2.97) ** | 2.43 (1.78 - 3.32) ** | 1 |  |  |
| Vascular atherosclerotic events (n=9,400) | 1.18 (0.85 - 1.65) | 1,99 (1.58 - 2.50)** | 1 | … |  |
| Death for all causes  ( n=11,117) | 1.55 (0.95 - 2.53) | 2.09 (1.46 - 3.00) ** | 1 | … |  |
| Combined MACE (n=9,065) | 1.31(1.01 - 1.71) * | 1.86 (1.54 - 2.24) ** | 1 | … |  |
| Non-CVD death | 1.64 (0.96 - 2.80) | 1.90 (1.27 - 2.85)** | 1 |  |  |
| ***Model 2 (adjusted),***  ***HR (95% CI)*** |  |  |  |  |  |
| Incident HF (n=11,117) | 1.09 (0.79 - 1.52) | 1.65 (1.31 - 2.07)** | 1 | … |  |
| Incident AF (n=10,600) | 1.83 (1.22 -2.75) ** | 2.21 (1.61 - 3.03) ** | 1 |  |  |
| Vascular atherosclerotic events (n=9,400) | 1.09 (0.78 - 1.53) | 1.86 (1.47 - 2.35)** | 1 | … |  |
| Death for all causes  ( n=11,117) | 1.38 (0.84 - 2.27) | 1.75 (1.21 - 2.53)** | 1 | … |  |
| Combined MACE (n=9,065) | 1.23 (0.94 - 1.60) | 1.80 (1.48 - 2.18) ** | 1 | … |  |
| Non-CVD death | 1.40 (0.81 - 2.42) | 1.53 (1.01 - 2.32)* | 1 |  |  |

– Model 1 (unadjusted): clusters only.
– Model 2 (adjusted): clusters + covariates.
For incident HF, no prior HF cases existed by inclusion criteria; adjusted models include a composite prior-CVD covariate (prior MI, stroke, PAD, or AF), weighted as 1 or 0, alongside diabetes and hypercholesterolemia.
For incident AF, prevalent AF cases were excluded; adjusted models include prior-CVD composite, diabetes, and hypercholesterolemia.
For vascular atherosclerotic events, prevalent MI, stroke, or PAD cases were excluded; adjusted models include prior-CVD composite, diabetes, and hypercholesterolemia.
For MACE, all prior CVDs were excluded; adjusted models include only diabetes and hypercholesterolemia.
For all-cause and non-CVD death, no CVD exclusions were made; adjusted models include the prior-CVD composite, diabetes, and hypercholesterolemia.
Abbreviations: AF = atrial fibrillation; CI = confidence interval; HF = heart failure; HR = hazard ratio; MI = myocardial infarction; PAD = peripheral artery disease; CVD = cardiovascular disease; MACE = major adverse cardiovascular events.

* p <0.05; ** p<0.005

**Figure S1. Kaplan-Meier survival analysis for incident events stratified by clusters.**


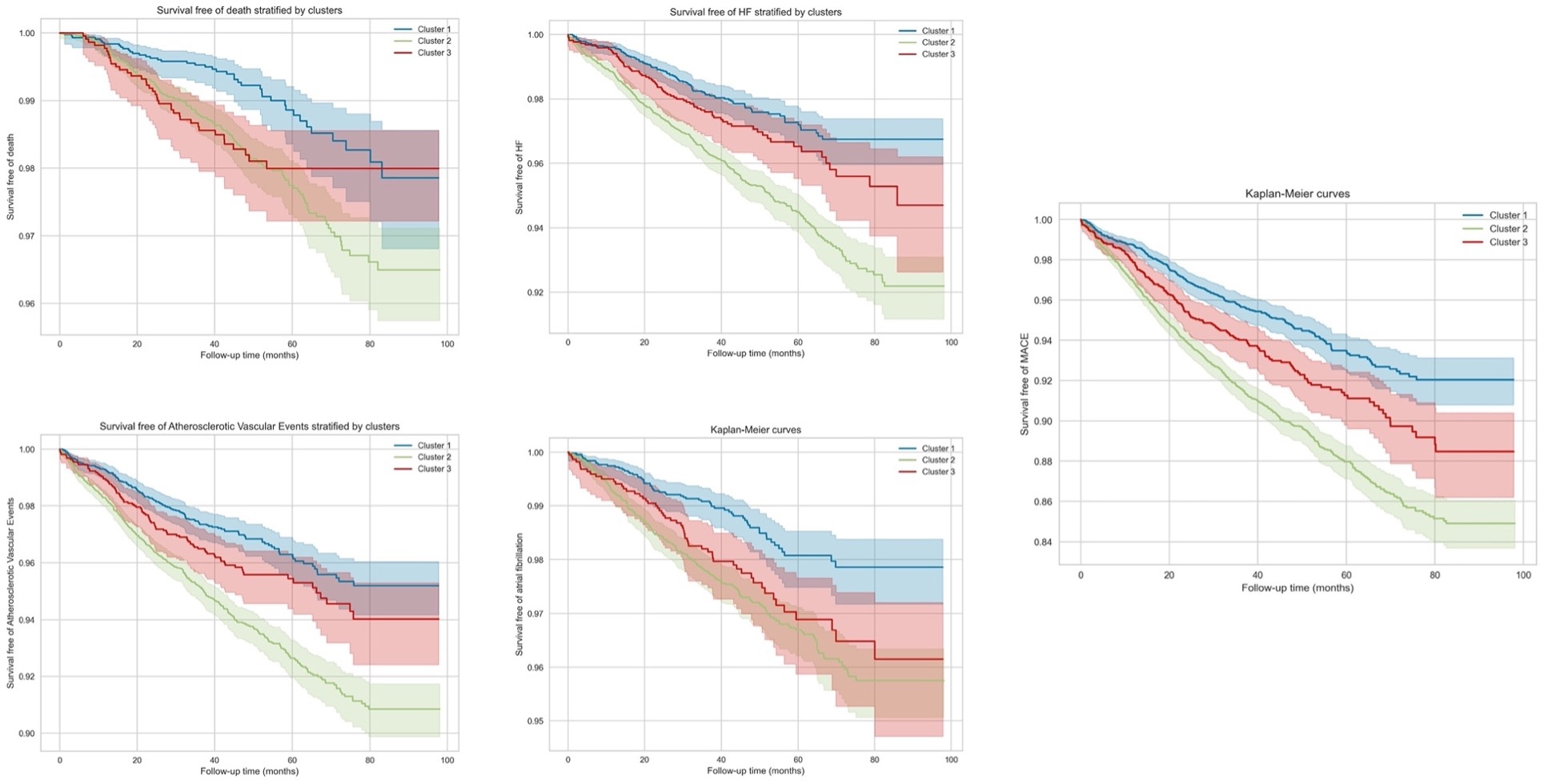


**Figure S2. Associations between CMR metrics and clustering - sensitivity analysis (prevalent conditions are excluded).**


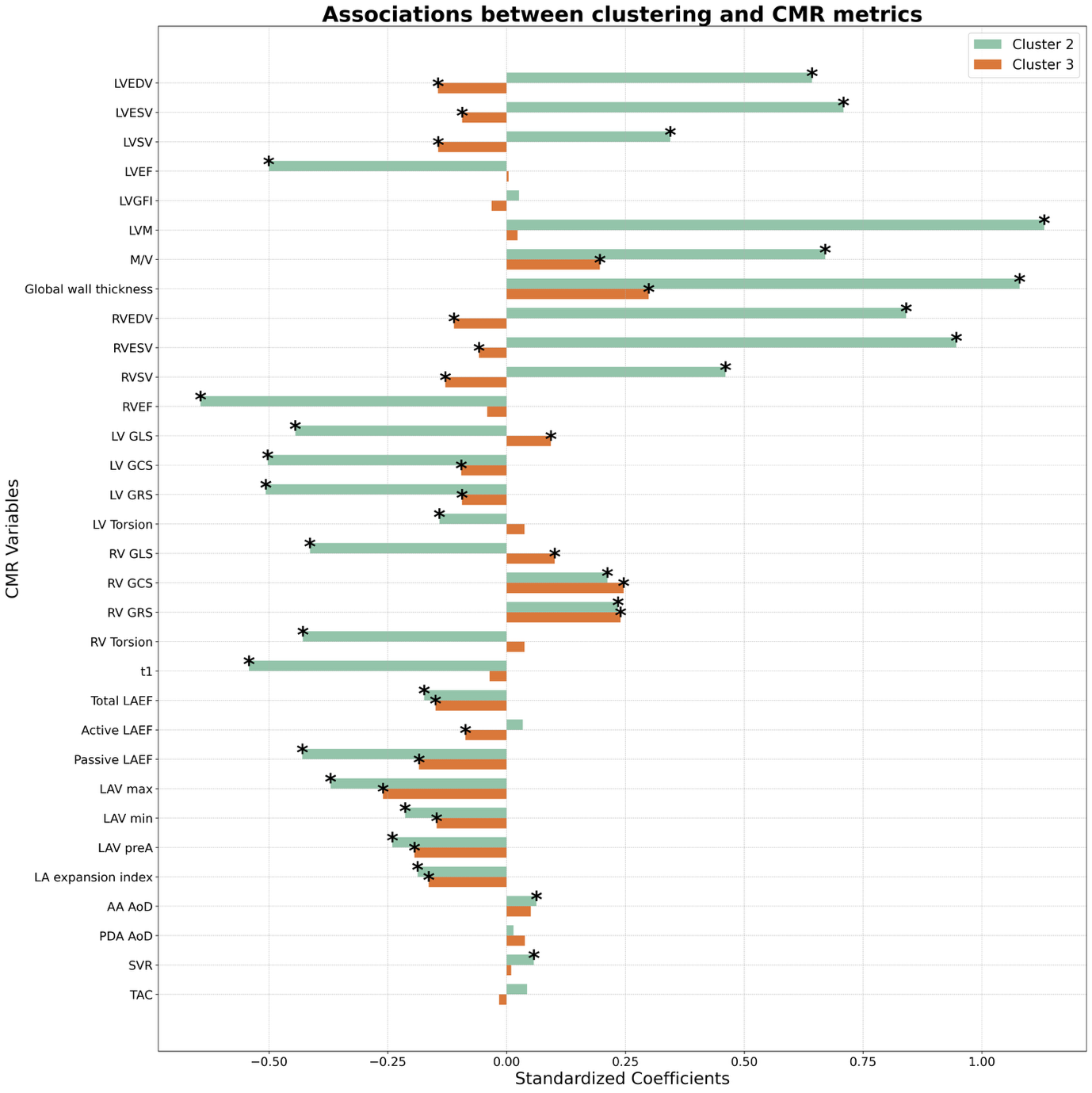


This figure illustrates the associations between CMR-derived metrics and clustering, comparing cluster 2 and cluster 3 to the lowest-risk reference group (cluster 1). The bars represent standardised beta coefficients from regression models, indicating the magnitude and direction of changes in each CMR metric when comparing clusters 2 and 3 to cluster 1. Positive coefficients suggest higher values of the respective CMR metric in clusters 2 or 3 compared to cluster 1, while negative coefficients indicate lower values relative to the reference group. Only significant associations after Bonferroni correction for multiple testing are displayed. Asterisks (*****) indicate statistically significant associations per each group.

**Figure S3. Associations between CMR metrics and clustering - sensitivity analysis (cardiomyopathy cases are excluded).**


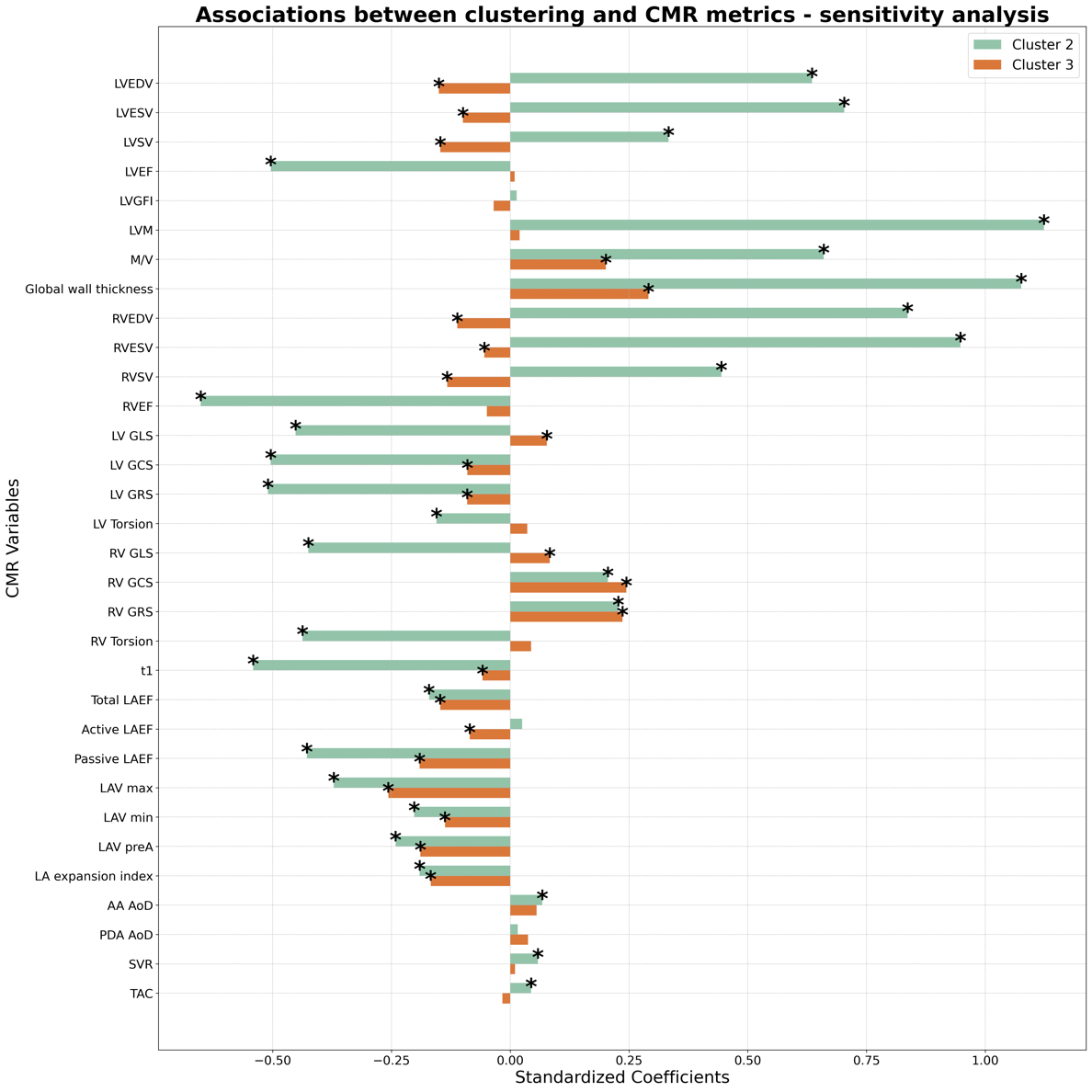


This figure illustrates the associations between CMR-derived metrics and clustering, comparing cluster 2 and cluster 3 to the lowest-risk reference group (cluster 1). The bars represent standardised beta coefficients from regression models, indicating the magnitude and direction of changes in each CMR metric when comparing clusters 2 and 3 to cluster 1. Positive coefficients suggest higher values of the respective CMR metric in clusters 2 or 3 compared to cluster 1, while negative coefficients indicate lower values relative to the reference group. Only significant associations after Bonferroni correction for multiple testing are displayed. Asterisks (*****) indicate statistically significant associations per each group.

**Figure S4. Proportion of MACE risk mediated by CMR features across clusters - sensitivity analysis (cardiomyopathy cases are excluded).**

**
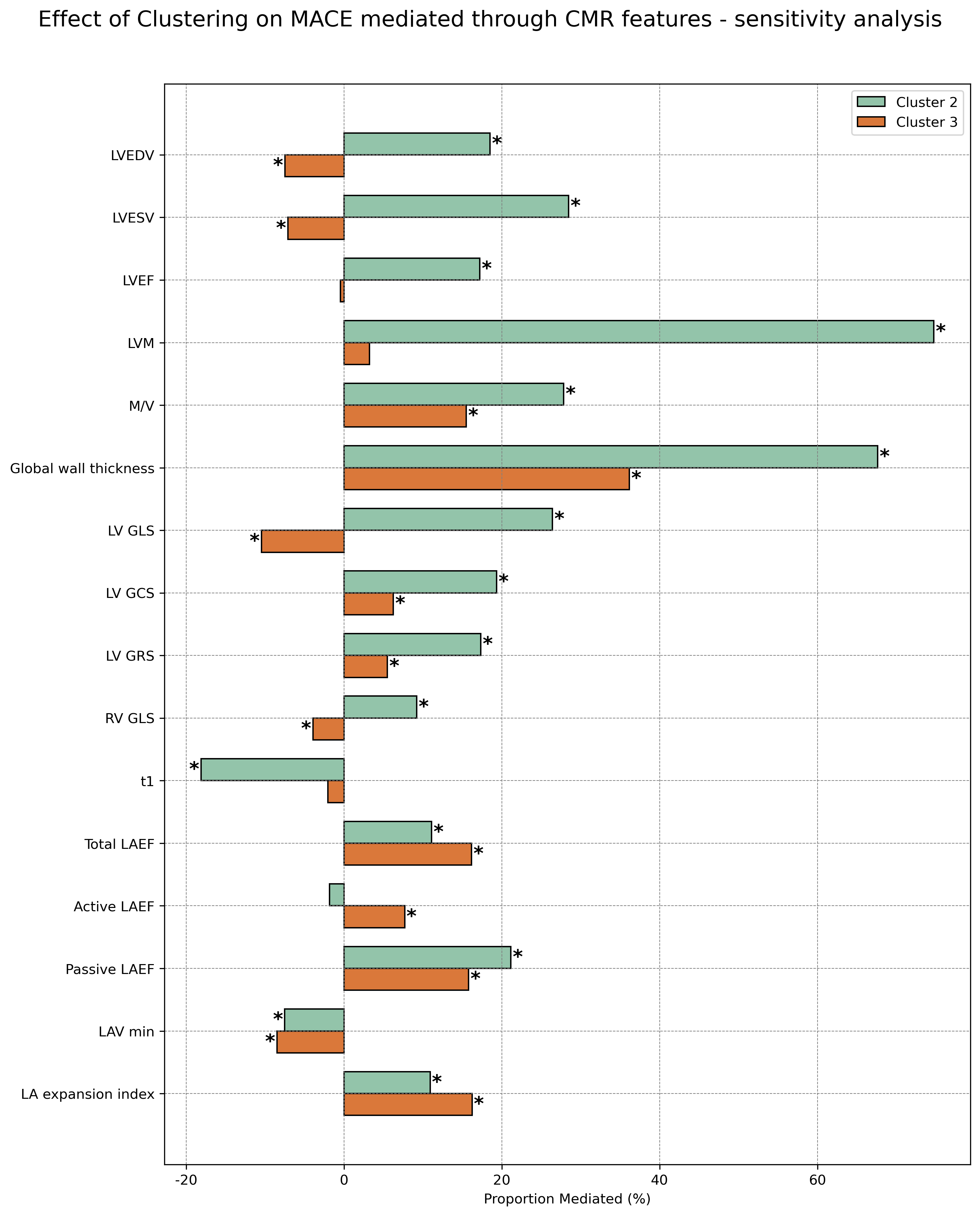
**

The figure shows the proportion of the total effect of clustering on the outcome mediated through CMR metrics, expressed as a percentage. Positive values indicate mediation, where the CMR variable explains part of the association between clustering and the outcome. Negative values suggest a suppressor effect, strengthening the direct association. Cluster 2 (higher-risk) and cluster 3 (intermediate-risk) are compared to cluster 1 (lowest-risk reference). Asterisks (*****) indicate statistically significant mediation effects.

**Figure S5. Proportion of death risk mediated by CMR features across clusters - sensitivity analysis (cardiomyopathy cases are excluded).**


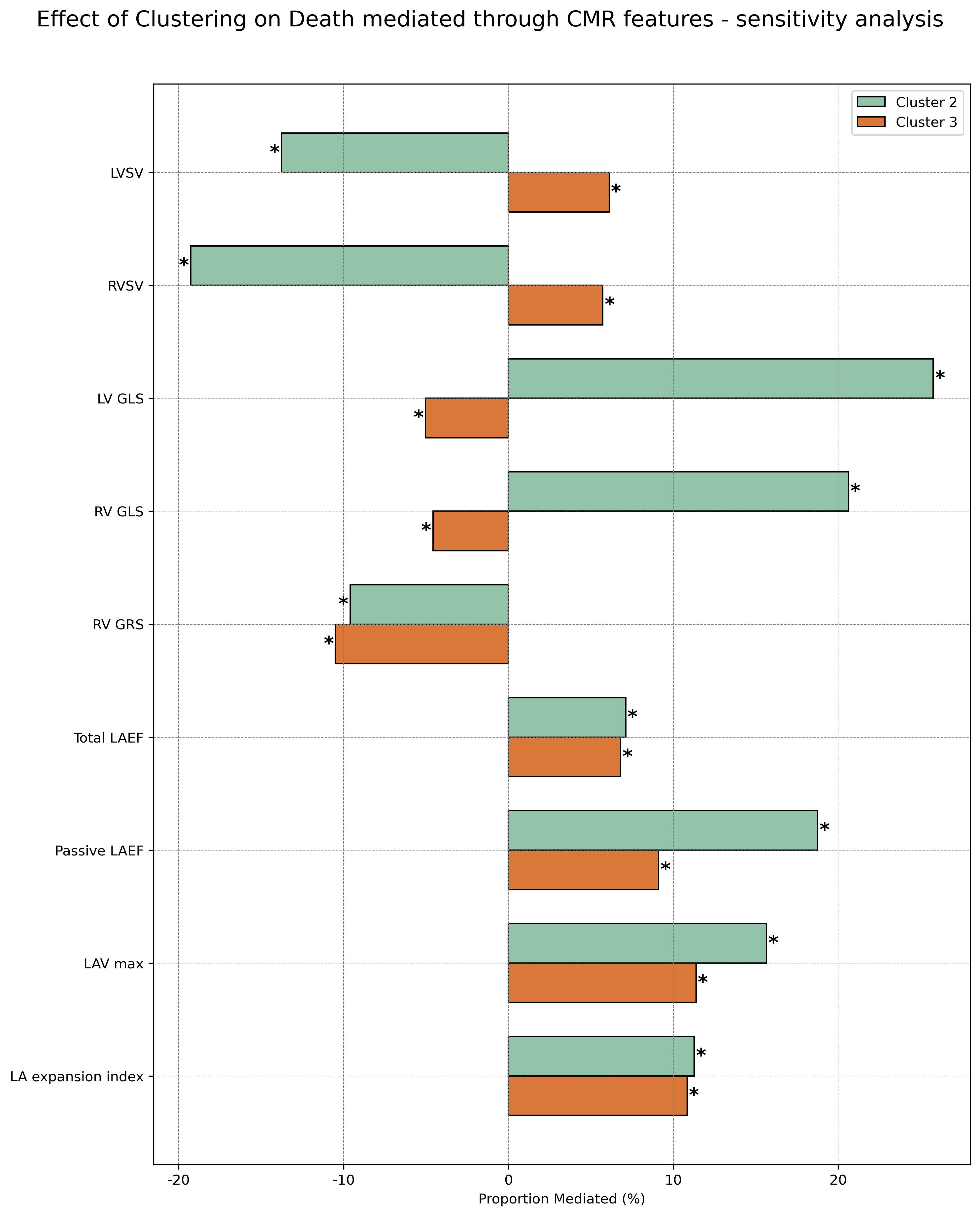


The figure shows the proportion of the total effect of clustering on the outcome mediated through CMR metrics, expressed as a percentage. Positive values indicate mediation, where the CMR variable explains part of the association between clustering and the outcome. Negative values suggest a suppressor effect, strengthening the direct association. Cluster 2 (higher-risk) and cluster 3 (intermediate-risk) are compared to cluster 1 (lowest-risk reference). Asterisks (*****) indicate statistically significant mediation effects.

**Figure S6. Elbow method for identifying the optimal number of clusters: FAMD + K-means approach -sensitivity analysis (temporal heterogeneity).**


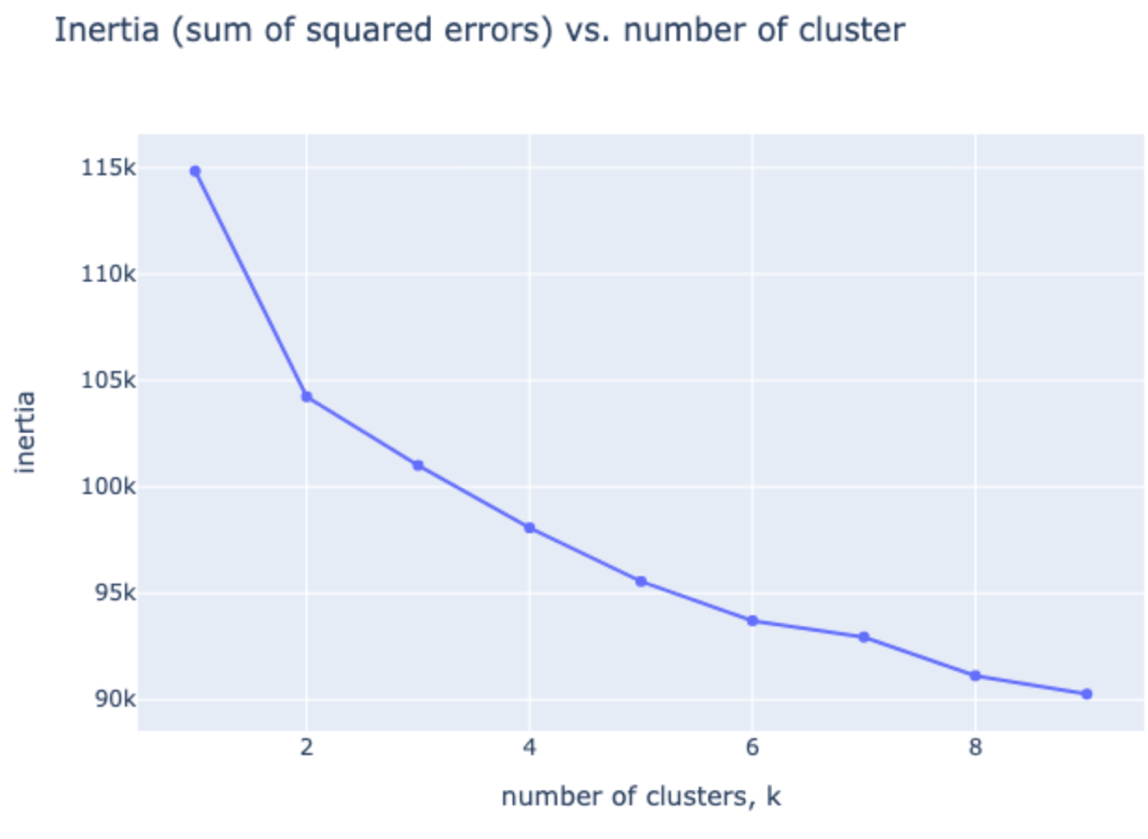


On the y-axis is the inertia for each value of clusters, k, (x-axis) n a given range (1-10). The optimal number of clusters that balances minimising the variance within clusters is identified as the point where an elbow-like bend with a lesser inertia value is observed. In this case, as confirmed by the knee locator, the elbow is at k=3, indicating an optimal number of clusters is three.

**Figure S7. Radar charts showing the top 32 features contributing to clustering - sensitivity analysis (temporal heterogeneity).**

**.**


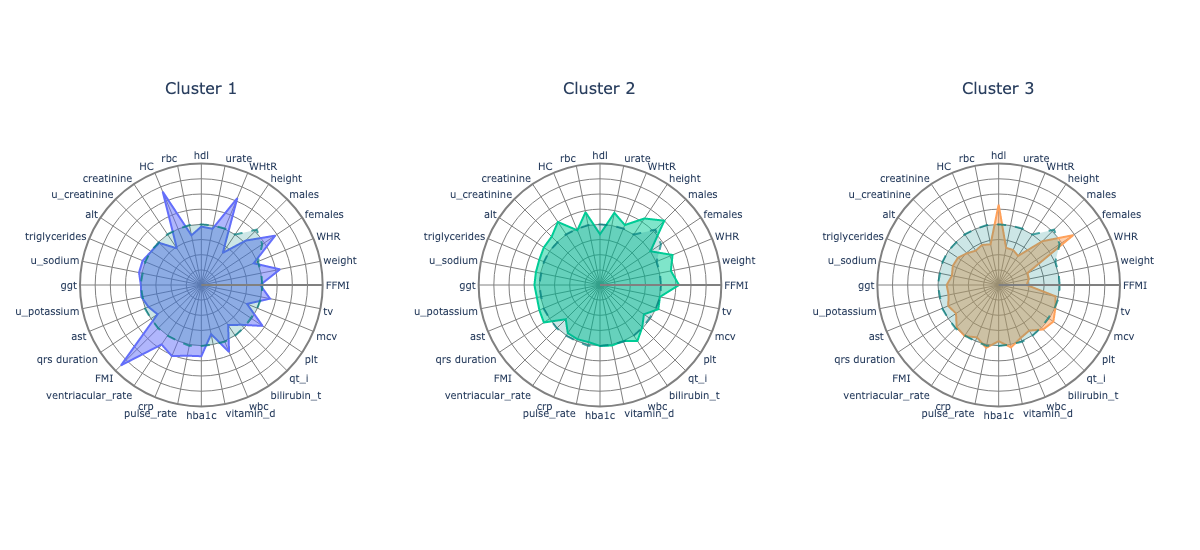


Radar plots summarising the top 32 (scaled) features contributing to clustering obtained from SHAP compared to their average value for the entire cohort (indicated by the dashed blue line). Abbreviations are explained in Table 1.

**Figure S8. Associations between CMR metrics and clustering - sensitivity analysis (temporal heterogeneity).**


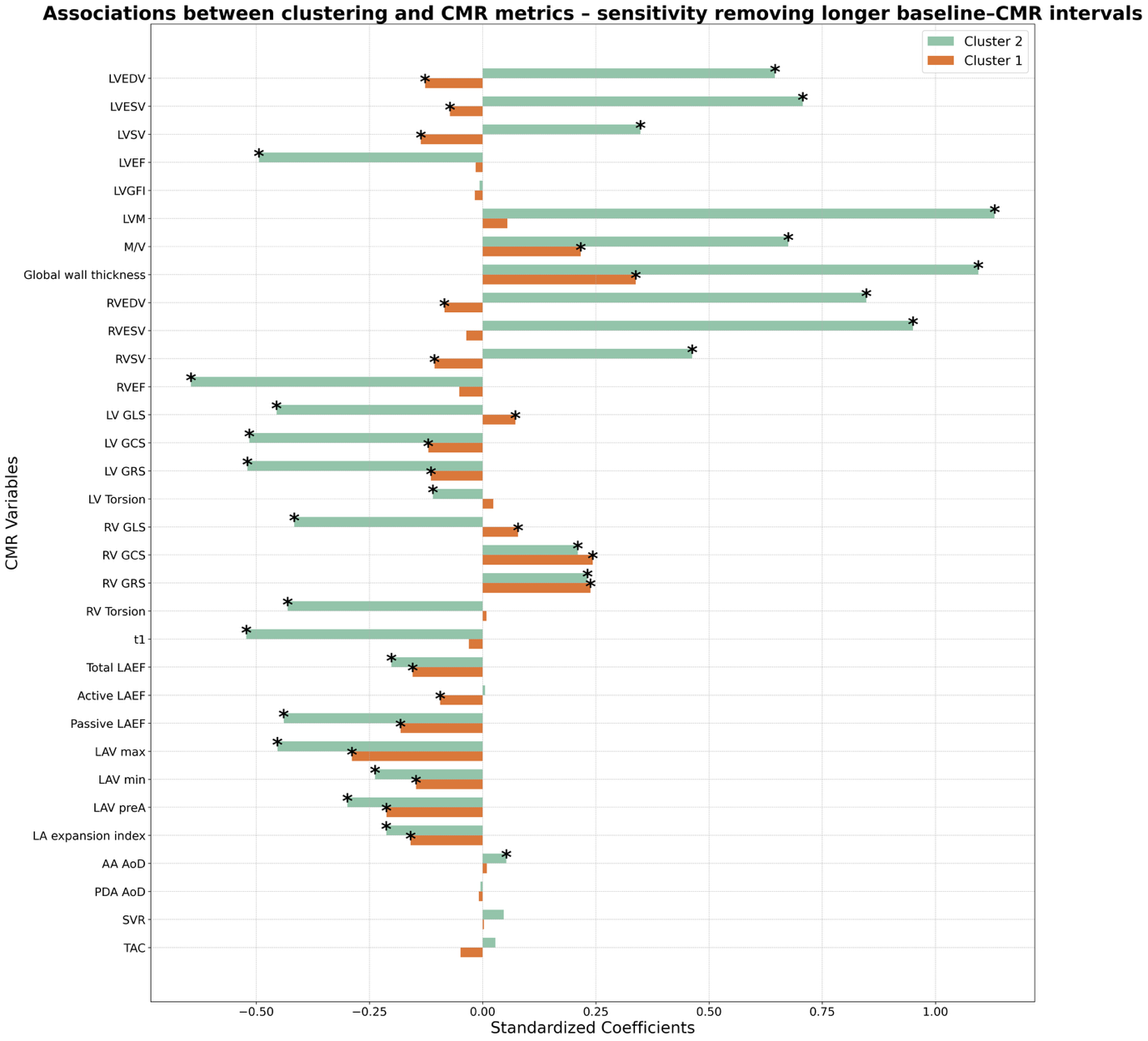


This figure illustrates the associations between CMR-derived metrics and clustering, comparing cluster 2 and cluster 1 to the lowest-risk reference group (cluster 3). The bars represent standardised beta coefficients from regression models, indicating the magnitude and direction of changes in each CMR metric when comparing clusters 2 and 1 to cluster 3. Positive coefficients suggest higher values of the respective CMR metric in clusters 2 or 1 compared to cluster 3, while negative coefficients indicate lower values relative to the reference group. Only significant associations after Bonferroni correction for multiple testing are displayed. Asterisks (*****) indicate statistically significant associations per each group.

**Figure S9. Proportion of MACE risk mediated by CMR features across clusters - sensitivity analysis (temporal heterogeneity).**


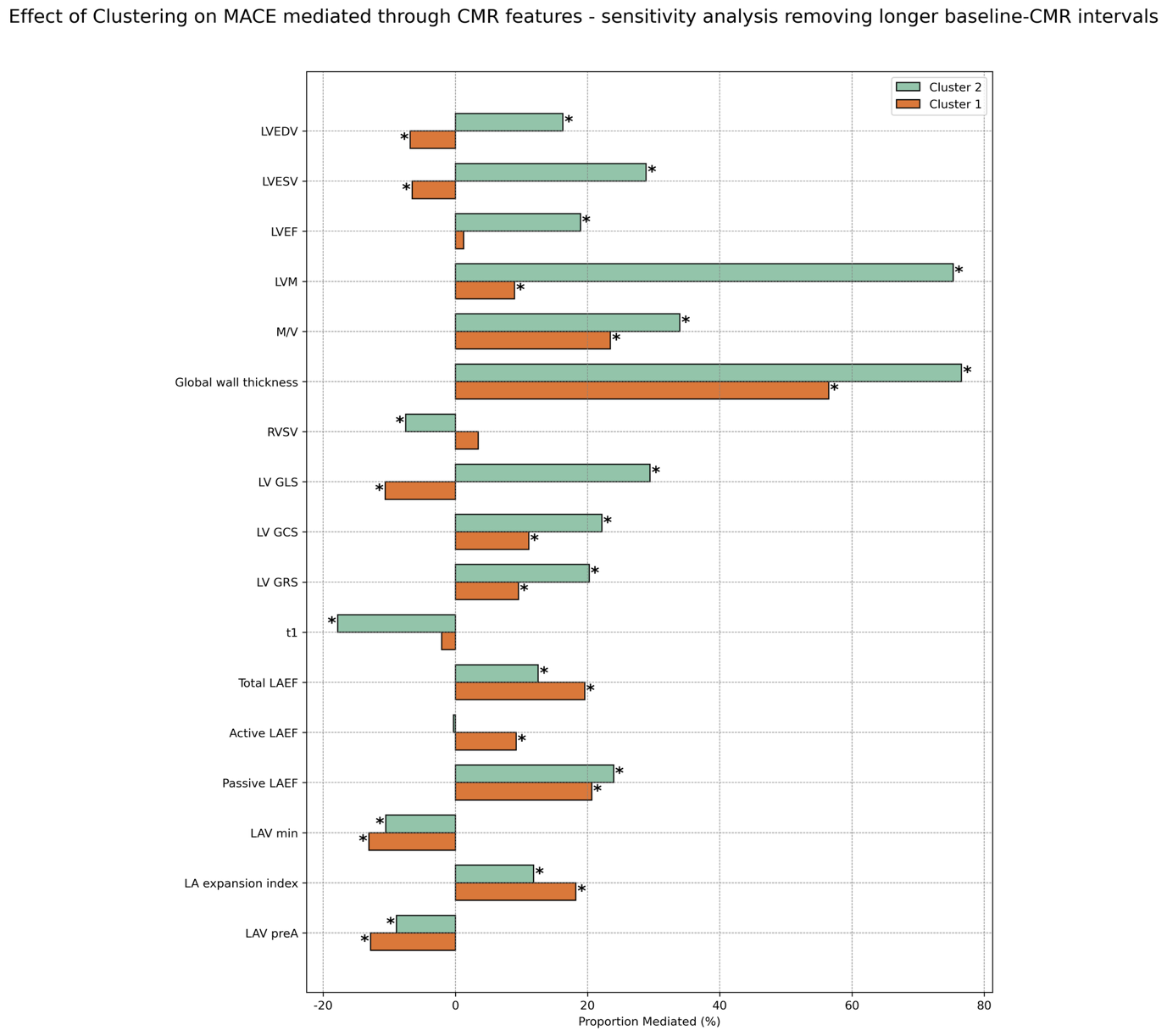


The figure shows the proportion of the total effect of clustering on the outcome mediated through CMR metrics, expressed as a percentage. Positive values indicate mediation, where the CMR variable explains part of the association between clustering and the outcome. Negative values suggest a suppressor effect, strengthening the direct association. Cluster 2 (higher-risk) and cluster 1 (intermediate-risk) are compared to cluster 3 (lowest-risk reference). Asterisks (*****) indicate statistically significant mediation effects.

**Figure S10. Proportion of AF risk mediated by CMR features across clusters - sensitivity analysis (temporal heterogeneity).**


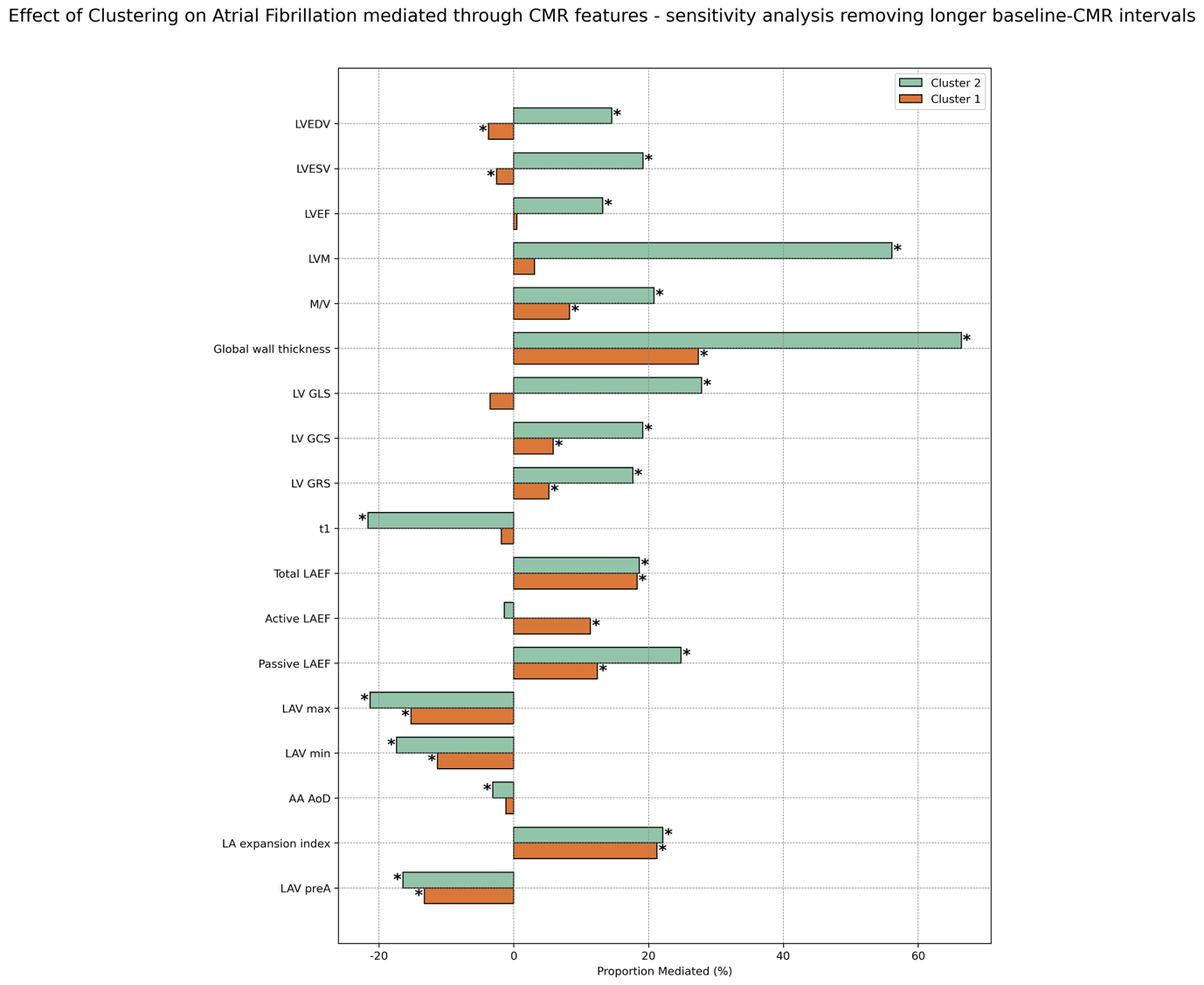


The figure shows the proportion of the total effect of clustering on the outcome mediated through CMR metrics, expressed as a percentage. Positive values indicate mediation, where the CMR variable explains part of the association between clustering and the outcome. Negative values suggest a suppressor effect, strengthening the direct association. Cluster 2 (higher-risk) and cluster 1 (intermediate-risk) are compared to cluster 3 (lowest-risk reference). Asterisks (*****) indicate statistically significant mediation effects.
